# High-throughput phenotyping reveals multiple drought responses of wild and cultivated Phaseolinae beans

**DOI:** 10.3389/fpls.2024.1385985

**Published:** 2024-09-27

**Authors:** Jon Verheyen, Stijn Dhondt, Rafael Abbeloos, Joris Eeckhout, Steven Janssens, Frederik Leyns, Xavier Scheldeman, Veronique Storme, Filip Vandelook

**Affiliations:** ^1^ Research Department, Meise Botanic Garden, Meise, Belgium; ^2^ Vlaams Instituut voor Biotechnologie (VIB), Agro-incubator, Nevele, Belgium; ^3^ Department of Biology, KU Leuven, Leuven, Belgium

**Keywords:** crop wild relatives, drought, high-throughput phenotyping, *Phaseolus*, stress, *Vigna*, water use efficiency

## Abstract

**Introduction:**

Although drought resistance of a plant may be achieved through morphological, structural, physiological, cellular, and molecular adaptations, most studies remain limited to quantifying the effect of drought on biomass.

**Methods:**

Using a highthroughput phenotypic imaging system, we evaluated the drought resistance of 151 bean accessions (Phaseolinae; Fabaceae) in an explorative approach, by quantifying five different traits simultaneously: biomass, water use efficiency (WUE), relative water content (RWC), chlorophyll content (NDVI), and root/shoot ratio. Since crop wild relatives are important resources for breeding programs, we analyzed both wild and cultivated accessions, most of which have never been evaluated for drought resistance before.

**Results:**

We demonstrate that the five traits are affected very differently by drought in the studied accessions, with significant correlations existing only between the biomass and WUE indicators (r=0.39), and between the RWC and NDVI indicators (r=0.40). When grouping accessions by subgenus or by species, large intraspecific and withinsubgenus variation was found. For this reason, we performed a cluster analysis, which grouped the accessions into five distinct clusters with similar response profiles. We also correlated the drought resistance for each accession to local climate variables at their original collection sites. The biomass, WUE, and RWC indicators were significantly correlated to annual precipitation (r=0.40, r=0.20, r=0.22, respectively), confirming that accessions from arid environments are generally more drought resistant.

**Discussion:**

Our results demonstrate that the drought resistance of Phaseolinae beans is a multifaceted characteristic and cannot be simply quantified through biomass. Furthermore, the broader knowledge of the drought resistance of the accessions studied here may prove an invaluable resource for future crop production.

## Introduction

Drought stress is one of the most important abiotic stresses affecting crop yield worldwide (Daryanto et al., 2016). Climate projections suggest that the Earth’s temperature will continue to rise throughout this century, leading to more frequent extreme climate events, including heatwaves, heavy precipitation, and drought (IPCC, 2021). Consequently, the frequency of severe drought episodes is expected to increase, posing a serious threat to crop cultivation. Therefore, investigating the drought resistance of cultivated food crops has become a paramount area of scientific research.

A plant’s response to drought stress is multifaceted and can be expressed in various ways. Several strategies can be distinguished in different plant species for coping with drought stress. One is drought escape, in which the plant is vulnerable to prolonged drought, but compensates for this with a short lifecycle that can be completed outside of the most severe drought periods. Another is drought evasion, where the plant avoids stress by restricting its water uptake or growth during periods of drought to avoid depleting soil moisture (Shantz, 1927). A third strategy is drought tolerance, where the plant has mechanisms to tolerate stress caused by drought. All of these strategies determine a plant’s drought resistance (Bandurska, 2022). Coping with drought is achieved through morphological, physiological, molecular, and structural adaptations. Although the capacity of a plant to resist drought is determined by all of these adaptations and the interactions between them, studies dealing with drought stress and plant cultivation focus mainly on the effect of drought on biomass, which is considered the most valuable trait for food production. However, multiple other ways exist to quantify plant responses to drought, which can cover different aspects of the drought response spectrum.

Evaluating the effect of drought on biomass is often based on measurements under drought stress compared to a control group. However, this approach presents challenges, as smaller plants may retain a higher biomass relative to their control group than larger plants (Iseki et al., 2018). One solution is to measure water use efficiency (WUE) instead of absolute biomass. A plant’s WUE is the amount of biomass produced per unit of water consumed (Briggs and Shantz, 1913). Since lower growth rates are associated with lower water consumption, WUE alleviates the bias towards smaller plants. In agricultural studies, WUE is therefore a more commonly used index (e.g., Anyia and Herzog, 2004).

Drought stress also affects plants by limiting the amount of water available to different tissues. A decrease in relative water content (RWC) lowers the water potential and can increase the temperature in tissues, which can lead to lower photosynthetic rates (Siddique et al., 2000). RWC has also been shown to influence the concentrations of pigments such as chlorophyll and carotenoids (González-Espíndola et al., 2024). Depending on a plant’s drought resistance, its water uptake and transpiration rates, and thus its RWC, will be affected differently under drought stress. For this reason, RWC can also be used as a trait for drought resistance screening (Schonfeld et al., 1988; Siddique et al., 2000; Belko et al., 2012). Several different methods to estimate RWC exist. The equation from Schonfeld et al. (1988) accurately calculates the RWC of plant tissues, but this method requires weighing the tissue before and after prolonged soaking and after oven drying, making it time-intensive and unsuitable for high-throughput purposes. Spectroscopic measurements, on the other hand, provide a more efficient approach. Water absorbs short-wave infrared light (900 nm – 2500 nm), and for this reason, the RWC of plant tissues can be correlated to the reflectance spectra at these wavelengths (Kim et al., 2015).

At the molecular level, one of the processes that is triggered by drought stress is an elevated production of H_2_O_2_, O_2_^-^ and other reactive oxygen species (de Carvalho, 2008). Within the chloroplasts, these lead to the degradation of chlorophyll, resulting in adverse effects (de Carvalho, 2008). A reduced relative chlorophyll content causes a lower photosynthetic rate (Fleischer, 1935) and has been associated with yield loss (Borrell et al., 2000). Through fluorescence measurements, Mathobo et al. (2017) demonstrated a decrease in chlorophyll content of *P. vulgaris* under different drought treatments. Vegetation reflects electromagnetic waves mostly in the near-infrared range (780 nm - 2500 nm), while chlorophyll strongly absorbs red light. Consequently, spectroscopic indices making use of these wavelengths can be used to estimate the relative chlorophyll content. The normalized difference vegetation index (NDVI) was originally proposed for remote sensing to non-destructively estimate the amount of green biomass in satellite imagery (Rouse et al., 1974). This index, which is calculated as the difference between near-infrared reflectance and red reflectance, divided by their sum, has since been adopted as an accurate estimator for relative chlorophyll content in phenotyping studies (Bell et al., 2004) and as an indicator of drought stress (e.g., Trapp et al., 2016; Condorelli et al., 2018; Javornik et al., 2023; Reddy et al., 2024).

Finally, characterizing the root system can give a more comprehensive understanding of the plant’s capacity to withstand drought conditions (Qi et al., 2019; Ma et al., 2021). Since an extensive, fast-growing root system can extract more moisture from the soil, plants with a large root system are often considered more drought resistant. To eliminate a bias towards naturally large plants, the ratio between root biomass and shoot biomass is often used as an indicator. For many crops, including *Phaseolus vulgaris*, it has been shown that investing more energy into the development of the root system relative to the shoot, results in a higher drought resistance compared to plants with a low root/shoot ratio (Haider et al., 2012; Sofi et al., 2018). Plants of *P. vulgaris* and *Vigna radiata* experiencing drought stress have also been shown to alter their development towards a higher root/shoot ratio (Sofi et al., 2018; Ikram et al., 2024). As such, this ratio can be used as a valuable predictor for drought resistance.

When economic, social, and infrastructural circumstances are considered, developing countries in Africa, Asia, and Latin America are deemed most vulnerable to future drought disasters (Carrão et al., 2016). A critical component of these nations’ food security relies on the production and consumption of beans from the Phaseolinae subtribe (Fabaceae family), including the economically important *Vigna* and *Phaseolus* genera. These legumes rank prominently in agricultural land allocation for food crops. They account for the second largest share in Central America, followed by a third place in Africa and a fifth place in South America and Asia (Food and Agriculture Organization of the United Nations, 2020). Most cultivated species within the Phaseolinae clade are restricted to the two genera *Vigna* and *Phaesolus*. The *Vigna* genus encompasses approximately 118 extant species, while *Phaseolus* comprises around 97 described species (WFO, 2022a, b). The most well-known food crops include the common bean (*P. vulgaris*), cowpea (*V. unguiculata*), runner bean (*P. coccineus*), Bambara groundnut (*V. subterranea*), mung bean (*V. radiata*), lima bean (*P. lunatus*), tepary bean (*P. acutifolius*), adzuki bean (*V. angularis*) and the black gram (*V. mungo*).

Most species that are currently cultivated are found in moderately to extremely wet climates, but several crop wild relatives from these genera live in semiarid to arid environments (Fick and Hijmans, 2017). Since crop wild genetic resources are useful tools in breeding programs (Dempewolf et al., 2014), the current study characterizes the degree of drought resistance for both wild and cultivated bean species. Apart from the study of Iseki et al. (2018), in which 69 *Vigna* accessions (including nine cultivated and 28 wild species) were screened, the degree of drought resistance of many wild bean species has not been previously investigated. The results of Iseki et al. (2018) indicated that several wild accessions were highly drought-resistant, including accessions belonging to *V. subramaniana*, *V. trilobata*, *V. vexillata* var. *ovata* and *V. aridicola*. They also demonstrated that different genotypes from the same species can exhibit highly variable levels of resistance (Iseki et al., 2018). For *Phaseolus*, no comparable large-scale studies have been performed to date.

The main aim of this explorative study was to expose different responses to drought stress in a wide diversity of wild and cultivated Phaseolinae beans. Due to the wide range of drought resistance mechanisms that can be expected in such an experiment, it is not sufficient to simply characterize drought tolerance. Rather, this study attempts to characterize drought resistance as a whole. To this end, we used five different drought resistance indicators derived from measurements of a high throughput phenotyping platform. The various plant traits upon which the resistance indicators in this study are based (i.e., biomass penalty, water use efficiency, relative water content, relative chlorophyll content, and root/shoot ratio), are all driven by different processes and are therefore expected to cover different areas of the drought resistance spectrum. In addition, given that wild plants adapt to local climate conditions, we tested whether the different drought stress indicators are related to climate variables of the locations where the accessions originally grew. Finding close correlations between the drought resistance indicators and climate conditions at the site of origin, would validate the approach of using high-throughput phenotyping in controlled conditions for large-scale drought resistance screening of bean crop wild relatives. We specifically addressed the following questions: (I) How do different indicator variables relate to each other?; (II) How much variation exists within and among subgenera?; (III) Are there groups of accessions with similar drought responses?; (IV) Do the drought resistance indicators correlate with the climate at the site of origin? We hypothesized that: (1) The drought resistance indicators would each show different responses under drought stress, proving that they each convey unique information, although we did expect that some indicators (e.g., RWC and NDVI) correlate more than others; (2) Accessions would group together according to their drought responses, as indicated by significant differences in the drought resistance indicators between groups; (3) Drought resistance indicator values would be correlated with climate at the original sampling location. If the latter hypothesis is confirmed, this would also provide robust support for the reliability of the experimental design.

## Materials and methods

### Phaseolinae accessions

The 151 Phaseolinae accessions studied included 8 genera (all of which were formerly considered part of either *Vigna* or *Phaseolus*), 12 subgenera and 65 species (for information on individual accessions, see **Supplementary Table 1**). All taxon names were checked against the World Flora Online database (WFO 2022a, b). Classification in subgenera followed Maréchal et al. (1978), and was updated with information from the WFO database (WFO 2022a, b). Of the used accessions, 23 are defined as cultivated and 128 as wild. Seeds of each accession were obtained from the Meise Botanic Garden collection, Belgium. The accessions were selected in such a way that they covered a wide taxonomic and ecological diversity, with a focus on *Vigna* species (including former *Vigna* genera) but supplemented with *Phaseolus* species. The accessions covered a large variety of local precipitation conditions, ranging from arid (< 200 mm annual precipitation) to extremely wet (> 2500 mm annual precipitation). Seeds had been stored in long-term storage conditions (-20°C and 15% relative humidity) for variable amounts of time, but all had high viability (> 80%) and vigor.

Climate data based on coordinates at collecting sites were retrieved from WorldClim using DIVA-GIS (Hijmans et al., 2001). WorldClim contains high-resolution climate data from the years 1970-2000, which is when most of our accessions were collected (Fick and Hijmans, 2017). WorldClim provides data on 19 different climate variables, many of which are highly correlated. Therefore, we selected five variables that were both intuitive and informative: isothermality (quantifies how large the day-to-night temperatures oscillate relative to the summer-to-winter [annual] oscillations), precipitation seasonality, temperature seasonality, annual mean temperature, and annual precipitation. Coordinates were derived from information on collecting location, whenever such information was sufficiently precise to derive coordinates. As such, climate data were derived for 118 ‘wild’ accessions (**Supplementary Table 1**).

### Drought treatment and soil moisture measurement

For each accession, ten seeds were scarified with a scalpel to break seed coat dormancy. Each seed was sown in a separate transparent plastic P12 pot (800 mL) containing potting soil (‘Potgrond voor Zaaien en Stekken’, Saniflor, pH 6.6, bulk density 0.301 kg/L, SOC 16%, CEC 66 cmol_C_/kg, based on peat, perlite, and fertilizer, detailed profile in **Supplementary Table 2**), supplemented with 3 g/L Osmocote^®^ fertilizer (Exact Standard 3-4M, Everris, **Supplementary Table 2**). The pots were then arranged on flooding tables in a greenhouse following a completely randomized design (**Supplementary Figure 1A**).

**Figure 1:**
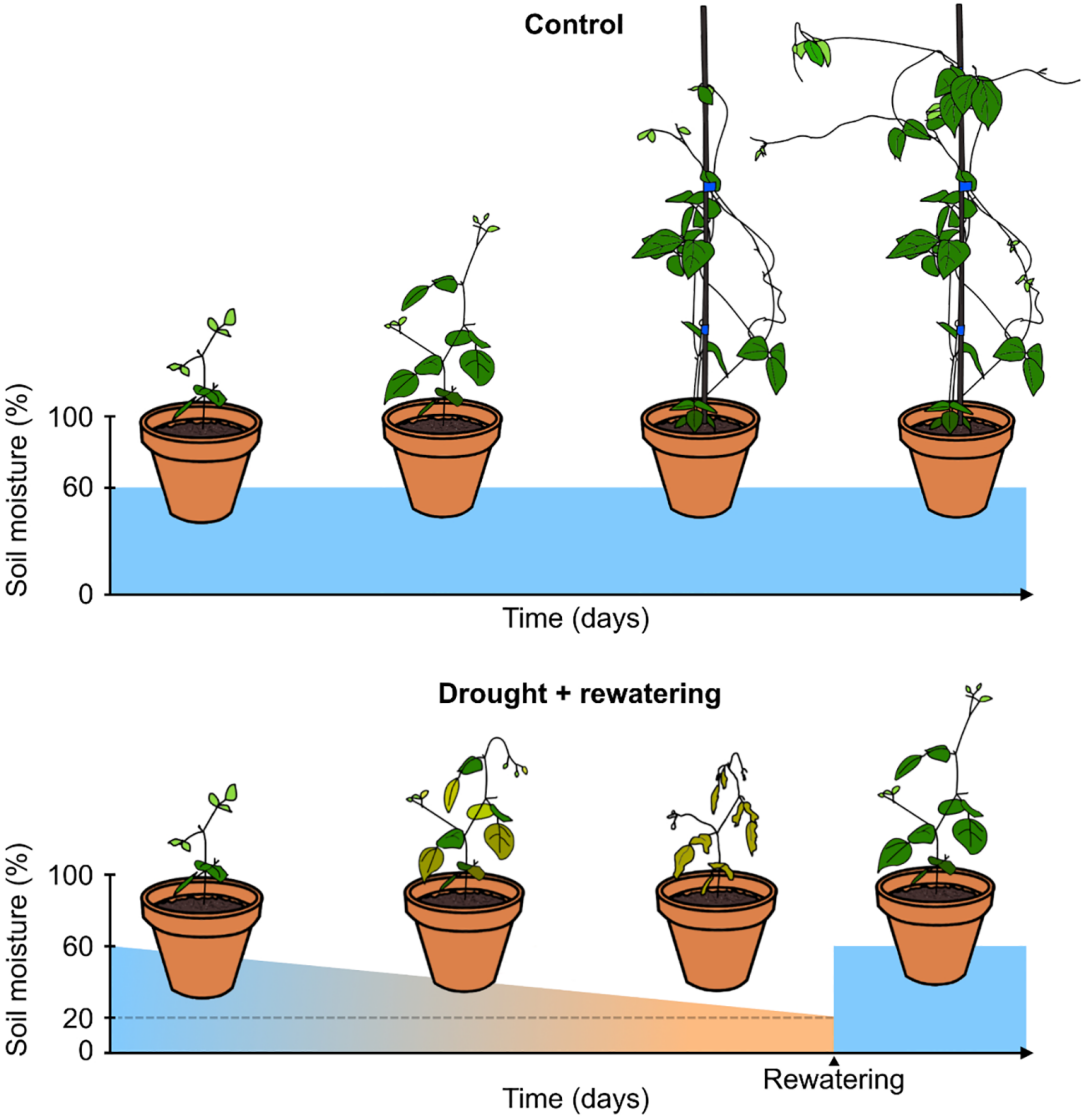
Watering scheme for drought treatment pots and control pots. Control pots were kept well-watered for a relatively constant soil moisture value of 60%. Pots under drought treatment were not watered until they reached a soil moisture value lower than 20%, at which point they were rewatered.

All pots were watered on these tables every 2-3 days to maintain constant soil moisture. Watering was done by flooding the tables completely, so that water could enter the pots through holes in the bottom (**Supplementary Figure 1B**). After watering, the tables were drained again, so that all excess water also drained from the pots. This ensured that pots were watered until field capacity and prevented waterlogging. The watering of all pots continued until 18 days after sowing (DAS). On this date, the drought treatment started for five of the ten pots from each accession, while the other five pots served as a control group (schematic representation in **Figure 1**). For every plant, data was collected on 18, 25, 29, 33, 36, 39, 43, and 46 DAS, once on each day. There were thus five technical replicates per accession, but no biological replicates in time. The control pots were watered every 1-2 days to maintain field capacity. The drought treatment involved a complete cessation of watering, resulting in a progressively increasing drought stress over time. Soil moisture was measured on the previously mentioned days, until the pots were almost completely dry, as indicated by a threshold value (lower than 20%) of an MMS-0 contactless moisture sensor (ACO) based on high-frequency dielectric shift. When this threshold was reached, pots were rehydrated until a soil moisture around field capacity was re-established, to allow for recovery of the plants. At 43 DAS, the final pots had dried out to the threshold value, were rewatered, and were allowed to recover for 3 more days, before the final measurements on 46 DAS.

A day/night cycle with 13 hours of light was provided by SON-T lamps with an approximate photosynthetic photon flux density of 250 µmol/m²s photosynthetically active radiation. During the growth of the plants, day and night temperatures in the greenhouse were kept at a minimum of 20°C and 18°C, respectively. A relative humidity of approximately 65% was maintained at all times. Detailed temperature and humidity data can be found in **Supplementary Figure 2**.

### Phenotypic imaging

On each of the measuring days, we passed all plants through a high-throughput imaging system located at the VIB Agro-Incubator, Nevele, Belgium. Imaging of the drought-treated plants lasted for the entire length of the drought treatment, which varied per pot since plants dried out at different rates. One final imaging session was performed for each drought plant after rehydration. Imaging of the well-watered accessions lasted for the duration of the entire experiment. For comparison with the drought treatment group, only the control data from the days when the corresponding drought treatment plants were rewatered, were used. After being placed on conveyor belts (**Supplementary Figure 1C**), each plant passed by RGB, multispectral, and hyperspectral cameras as well as a soil moisture measuring unit. Shoot biomass estimates were determined as the average projected area in side-view of the shoot on six images, with a 27° axial rotation of the plant between each image. These images were taken by an Allied Vision Prosilica GT 4905C RGB camera. A calibration plate (ColorChecker Classic) was used for color correction. The projected shoot area was subsequently calculated using a custom algorithm in the HALCON machine vision software (version 21.05, MVTec Software GmbH, 2021). This algorithm performs automatic segmentation of the shoot image through color space conversion and color filtering, and subsequent ridge detection and morphological operations. A binary mask is then produced for the shoot versus everything else, and the number of pixels representing the shoot is counted. The relationship between the real projected area in mm² and the number of pixels was determined by imaging a calibration plate. The linear relationship between the projected side area and the aboveground biomass (R² = 0.83, **Supplementary Figure 3**) was determined in a preliminary experiment based on 20 accessions with 8 to 10 plants for each accession using the *reg* procedure in SAS Studio (version 5.2, SAS Institute Inc, 2019).

An identical RGB camera in bottom-view and a similar algorithm were used to determine the projected area of the peripheral roots on the bottom of the transparent pots as a proxy for root biomass. The algorithm first detects the area of interest (the pot), and then converts the image from raw to RGB to HSV color format. The image is then filtered using Fast Fourier Transforms, bandpass, and high pass filters to remove light fluctuations and scratches. The edges of the roots are then detected using built-in HALCON operators, and the segmented number of pixels is again converted to the real projected area. The reflectance of the plants at the wavelengths 1417 nm and 1528 nm (based on Kim et al., 2015) for the estimation of RWC was determined by a Specim SWIR-CL-400-N25E hyperspectral camera (1000-2500 nm, with a spectral resolution of 3 nm) in top view (Kim et al., 2015). NDVI values were obtained from a multispectral FluxData FD-1665 3CCD camera containing two monochrome cameras for 680 nm and 800 nm. All experimental metadata, images, and phenotypic measurements were managed by the PIPPA software (version 0.14.20, VIB, 2021).

### Calculating drought resistance indicators

For each bean accession, four drought resistance indicators were determined based on aboveground biomass, WUE, RWC, and NDVI. In addition, we calculated the root/shoot ratio which may be indicative of how well plants are adapted to drought. The biomass penalty was calculated as the proportion of aboveground biomass lost, due to the drought treatment, compared to the control group, averaged over the plants belonging to the same accession and undergoing the same treatment (**Supplementary Figure 4A**). Since the relationship between biomass and projected area was shown to be linear (**Supplementary Figure 3**), we calculated the biomass penalty directly from projected shoot area as [(area_projected,control_ – area_projected,drought_)/area_projected,control_]. We used the biomass estimates of the drought plants after rewatering and the corresponding control plants, for the most accurate comparison, avoiding drought-induced leaf rolling. A low biomass penalty indicates a more drought-resistant accession.

For the indicator based on WUE, we followed the changes in WUE of the plants under drought stress. Since the pots did not receive any water during drought stress, we used the decrease in soil moisture as a proxy for the amount of water consumed by the plants. WUE values of the plants for each time interval, as estimated with the projected shoot area as (area_projected,t2_ – area_projected,t1_)/(soil moisture_t1_ – soil moisture_t2_), were plotted against the mean soil moisture value over that time interval ((soil moisture_t1_ + soil moisture_t2_)/2, further simply denoted as the soil moisture value). A positive WUE value for a chosen time interval indicates an increase in projected side-view area over that time interval and thus plant growth, while a negative value indicates a decrease and thus plant wilting. WUE values were plotted against soil moisture for each plant belonging to the same accession and a constrained generalized additive model (CGAM) was fitted for each accession using the *cgam* package in R (version 1.17, Liao and Meyer, 2019), assuming a smooth increasing curve with three knots and a gaussian distribution (**Supplementary Figure 4B**). The soil moisture value at which the plants ceased growing and started wilting could be found as the intercept of the fitted curve with the x-axis (WUE = 0). This x-intercept, the soil moisture at wilting, served as our WUE-based indicator. A low value for this indicator implies that plants of the chosen accession only started wilting relatively late into the progressively worsening drought stress, thus suggesting a drought-resistant accession.

The third drought resistance indicator, the soil moisture at leaf desiccation, based on RWC, was determined as the soil moisture value at which relative water content starts decreasing significantly. RWC index values (reflectance_1528 nm_/reflectance_1417 nm_) (Kim et al., 2015) were plotted against their corresponding soil moisture values and a linear-plateau regression model was fitted with the Levenberg-Marquardt algorithm from the *minpack.lm* package in R (version 1.2-4, Elzhov et al., 2023) using the *SSlinp* self-starting function from the *nlraa* package (version 1.9.3, Miguez, 2019). The junction point between the two linear sections of the plateau regression indicated the soil moisture at which the RWC started decreasing, indicative of leaf desiccation (**Supplementary Figure 4C**). A low value implies that the plants’ leaves started losing their water content relatively late into the drought stress, suggesting a drought-resistant accession.

A fourth indicator, based on NDVI, was equivalent to the indicator based on RWC, but was derived from the level of chlorosis presumed to be related to varying soil moisture levels. The NDVI values [(reflectance_800 nm_ – reflectance_680 nm_)/(reflectance_680 nm_ + reflectance_800 nm_)] (Rouse et al., 1974) were plotted against the corresponding soil moisture values (**Supplementary Figure 4D**) and a linear plateau regression model was fitted as described before. The soil moisture value at which NDVI started dropping significantly was determined as the junction point of this regression. A low value for soil moisture at chlorosis implies a drought-resistant accession.

Finally, the root/shoot ratio was calculated as the average area_projected,root_/area_projected,shoot_ ratio of all plants of the same accession following the same treatment on 18 DAS (**Supplementary Figure 4E**). We only used measurements before the onset of the drought treatment, since measurements of the projected root area in pots under drought were unreliable. A high root/shoot ratio signifies plants that invest a relatively large amount of their growth into deeply penetrating roots relatively early in their development, implying a potentially greater capacity to resist drought stress.

### Statistical analyses

All analyses were performed with the R statistical software (version 4.2.2, R Core Team, 2022) in RStudio (version 2022.07.2 build 576, RStudio Team, 2022) or SAS Studio (version 5.2, SAS Institute Inc, 2019). Descriptive statistics, including mean, standard deviation, variance, and range were calculated for all five drought resistance indicators (n_accessions_ = 151). Spearman rank correlations were estimated between all indicator variables. These analyses were performed using the R base package.

To compare the proportion of within-subgenus variability and between-subgenus variability to the total variability, a mixed model analysis was performed on each indicator with subgenus as random effect. In addition, heterogeneous within-subject variability was modelled by estimating a different residual variance for each subgenus. This was done for each drought resistance indicator separately. Subgenera with three or fewer accessions were omitted from this analysis. Mixed model analysis was performed with the mixed procedure from SAS/STAT in SAS Studio. For species-wise comparison, due to the high number of species sampled for this study, there were only a few species with enough accessions to statistically compare their drought resistance. Nonetheless, since the drought resistance of cultivated species may be of general interest for breeding, a principal component analysis (PCA) was performed including only the cultivated bean species (cultivated accessions + wild accessions from the same species). All PCAs were performed using the *ggbiplot* (version 0.6.2, Vu, 2011), and *ggplot2* (version 3.4.2, Wickham, 2016) R packages.

All 151 accessions were clustered by the five drought resistance indicators with the Ward clustering method based on Manhattan distances using the *dist* and *hclust* function from the R *stats* package. The tree was subsequently cut in five clusters using the *cutree* function from the *dendextend* package (version 1.17.1, Galili, 2015). The number of clusters was based on the scree plot showing the within-cluster sum of squares (WSS) changes, using the *fviz_nbclust* function from the *factoextra* (version 1.0.7, Kassambara and Mundt, 2020) package. Visualization of the tree was done with the same package. Boxplots were generated by cluster membership.

A second PCA was performed with all 151 accessions to visualize the relationships between the different drought resistance indicators. We visualized the distribution of the different clusters obtained from the cluster analyses and wild versus cultivated accessions on the first two PC axes. Given that the data of the different indicator values covered similar ranges, no data transformations were performed prior to PCA analyses.

An RDA analysis was performed using the R *vegan* package (version 2.6-4, Oksanen et al., 2022) to visualize how climate conditions at the site of origin relate to the drought resistance indicator variables. The analysis was carried out with the 118 accessions for which climate data were available (**Supplementary Table 1**). As mentioned before, five climate variables (isothermality, precipitation seasonality, temperature seasonality, annual mean temperature, and annual precipitation) were included in the analysis together with the five drought resistance indicator variables. The full RDA model was statistically significant (P < 0.001, permutation test: #permutations = 999).

## Results

### Drought treatment and indicator variables

The number of DAS on which each plant in the drought treatment group reached the threshold soil moisture value of 20%, are listed in **Supplementary Table 1**. For each day of measuring, the number of plants that reached the threshold value on that day is shown in a histogram in **Supplementary Figure 5**. Mean values, ranges, and standard deviation for the five drought resistance indicators are listed in **Table 1**. The drought resistance as expressed by WUE was significantly positively correlated to the biomass penalty (r = 0.39; P < 0.001) (**Table 2**, **Supplementary Figure 6**). RWC was significantly positively correlated with NDVI (r = 0.40; P < 0.001). All other correlations were not significant after Bonferroni correction (k = 10).

**Table 1:** Descriptive statistics for all five moisture response indicators.

Indicator	Meaning of indicator	N	Min	Max	Mean	SD	Var
Biomass penalty	(Shoot biomass control – shoot biomass drought)/shoot biomass control	151	0.01	0.94	0.62	0.18	0.033
WUE indicator	Soil moisture value at which WUE = 0	151	0.23	0.47	0.34	0.05	0.003
RWC indicator	Soil moisture value at which RWC decreases significantly	151	0.13	0.64	0.31	0.08	0.007
NDVI indicator	Soil moisture value at which NDVI decreases significantly	151	0.21	0.65	0.39	0.096	0.009
Root/shoot ratio	Root biomass divided by shoot biomass at start of the experiment	151	0.01	0.4	0.78	0.06	0.004

N, number of accessions included; Min, minimum value; Max, maximum value; Mean, mean value across accessions; SD, ± 1 standard deviation; Var, variance.

**Table 2:** Spearman rank correlations (rho) between five moisture response variables across all accessions (n = 151).

Indicator	Biomass penalty	WUE indicator	RWC indicator	NDVI indicator	Root/shoot
Biomass penalty	1				
WUE indicator	0.40**	1			
RWC indicator	0.21*	0.28**	1		
NDVI indicator	-0.10	0.05	0.39**	1	
Root/shoot ratio	-0.16	-0.08	-0.16*	-0.20*	1

Asterisks indicate p-values (* < 0.05, ** < 0.001).

### Variation within and among subgenera and species

**Figure 2:**
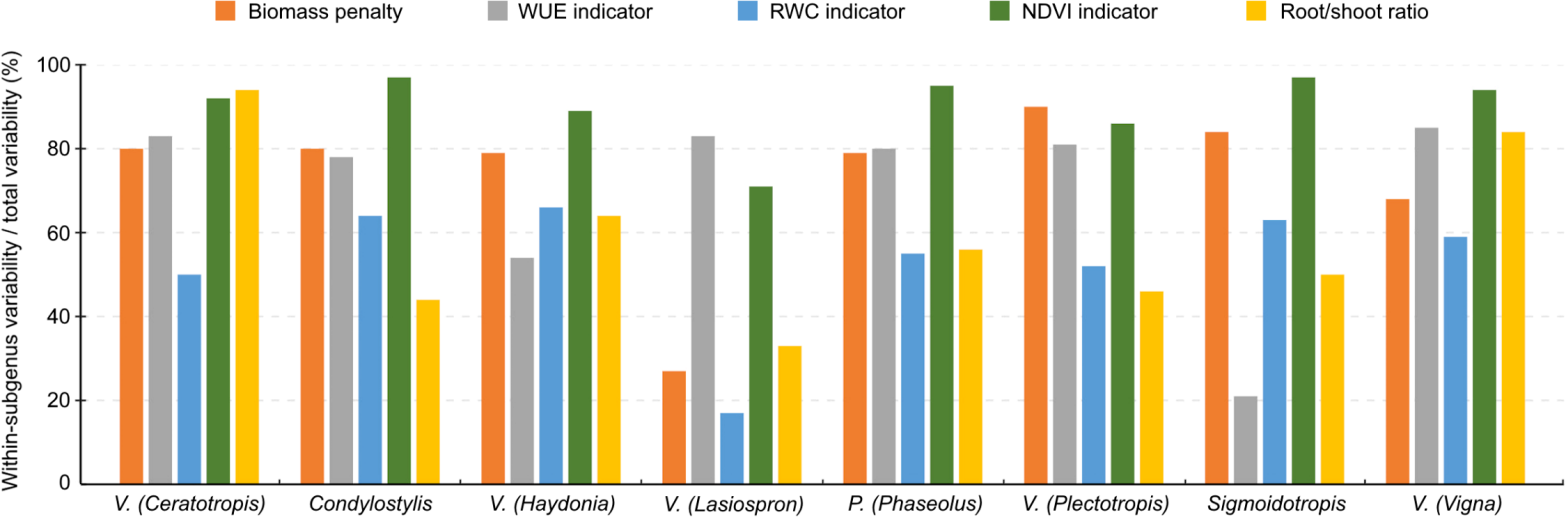
The proportion of within-subgenus variability/total variability in the five drought response indicator values for different subgenera. These were obtained using a mixed model analysis with subgenus as random effect. The heterogeneous within-subject variability was modelled by estimating a different residual variance for each subgenus. Subgenera with three or fewer accessions were omitted.

Within all subgenera except *Lasiospron*, most variation in the drought resistance indicators was observed to be within-subgenus variation (**Figure 2**; **Supplementary Table 3**). Between subgenera, some clear differences were also observed (**Supplementary Figure 7**). The mean values of indicators based on WUE, RWC and NDVI were highest in *Ceratotropis* (0.38 ± 0.05; mean ± SD), *Sigmoidotropis* (0.48 ± 0.07) and *Condylostylis* (0.50 ± 0.10) respectively, while they were always lowest in *Plectotropis* (0.31 ± 0.05; 0.25 ± 0.07 and 0.33 ± 0.06, respectively). The mean indicator based on biomass penalty was highest in *Lasiospron* (0.82 ± 0.06) and lowest in *Sigmoidotropis* (0.41 ± 0.21). There was, however, a great amount of variation in biomass penalty within several subgenera (**Supplementary Table 3**; **Supplementary Figure 7A**). The mean root/shoot ratio was highest in *Ceratotropis* (0.15 ± 0.12) and lowest in *Lasiospron* (0.04 ± 0.02). Especially for the indicators based on NDVI and biomass penalty, most variation was observed within subgenera (**Supplementary Table 3**). For the species-wise comparison of cultivated species, we found large intraspecific variations, despite the low number of sampled accessions for most species (**Supplementary Figure 8**). Nonetheless, each species does appear to possess a distinct drought resistance profile.

### Grouping bean accessions with similar drought responses

**Figure 3:**
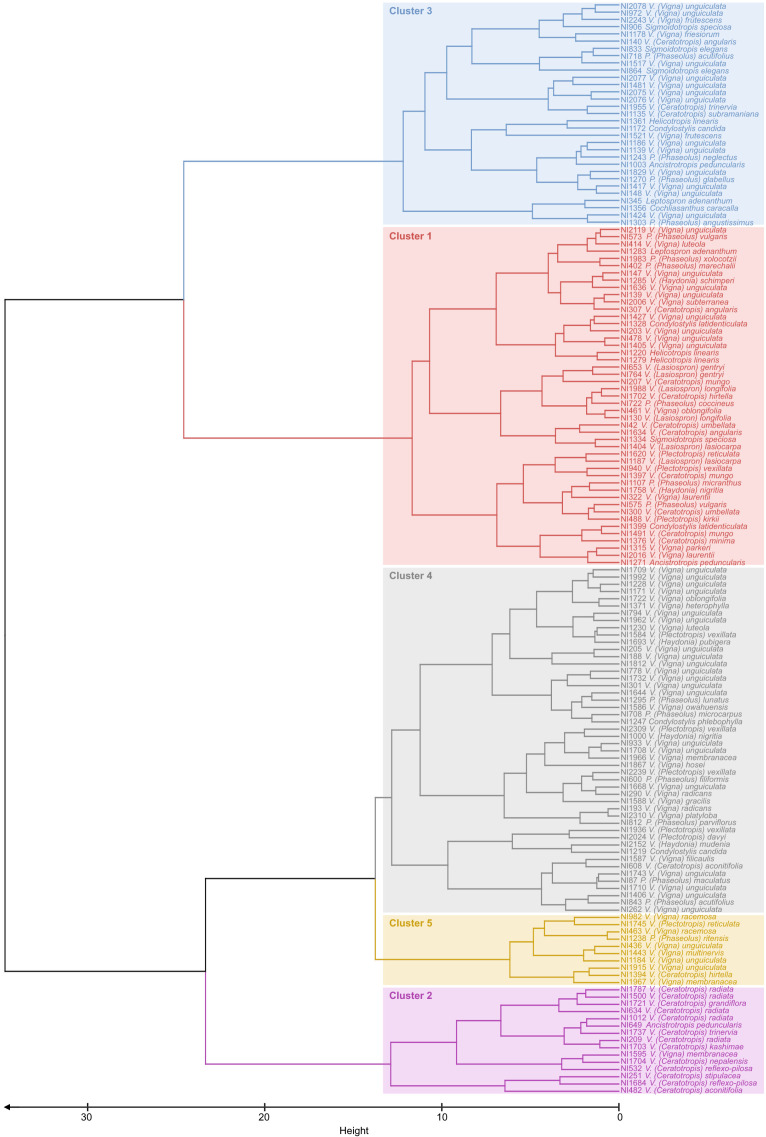
Dendrogram obtained by clustering analyses on 151 bean accessions with data for five drought response indicators. The Ward clustering method was used based on Manhattan distances. The tree was cut into five clusters, grouping accessions with similar drought responses.

**Figure 4:**
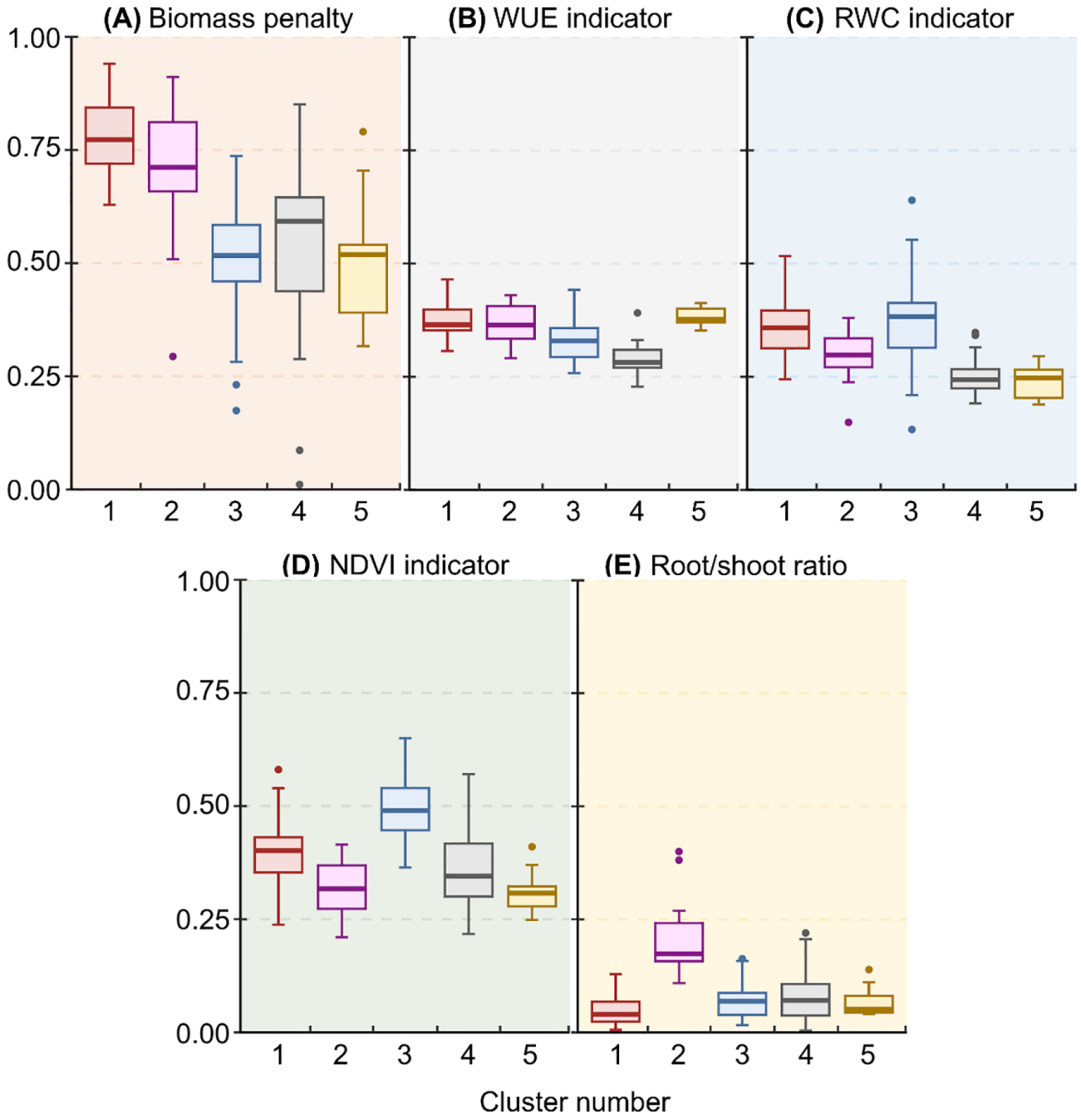
Boxplots of median drought response indicator values for **(A)** biomass penalty, **(B)** WUE, **(C)** RWC, **(D)** NDVI, and **(E)** root/shoot ratio, generated by a cluster analysis on 151 bean accessions.

Cluster analyses enabled us to group accessions into five clusters of accessions with similar drought responses within clusters (**Figure 3**). Clusters 1 and 2 (**Figure 3**) were characterized by high values for biomass penalty (**Figure 4A**), which may indicate that these are tall plants whose growth is considerably reduced by drought. Accessions in cluster 2 also showed a higher root/shoot ratio than other accessions (**Figure 4E**), indicating that their growth is affected by drought despite a higher root/shoot ratio. Accessions in cluster 3 had a high indicator value for RWC and NDVI (**Figures 3**, **4C, D**), which means that the response was mainly visible in the form of reduced photosynthesis. The accessions in cluster 4 had low values for the WUE and RWC indicators (**Figures 3**, **4B, C**), meaning they responded better to drought than accessions in other clusters. Finally, accessions in cluster 5 had high values for the WUE indicator (**Figures 3**, **4B**) and low values for all other indicator variables.

**Figure 5:**
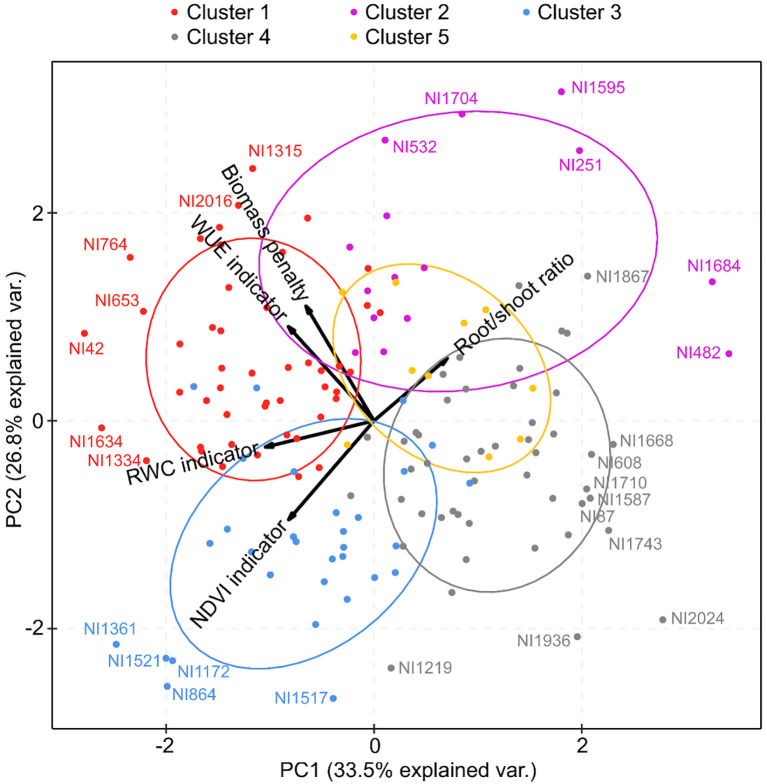
Principal component analysis (PCA) visualizing the relationships between the five different drought response indicators. The analysis was performed with all 151 accessions. The five clusters obtained from the cluster analysis are represented with different colors. Only the two first PCA axes are shown, in accordance with the Kaiser criterion.

A total of 151 bean accessions were included in the PCA (**Figure 5**). The first two PCs explained 33.5% and 26.8% of the variance. The PCA confirmed the positive correlation between the WUE indicator and the biomass penalty and between the RWC and NDVI indicators. It further suggests a negative correlation between the root/shoot ratio and the NDVI indicator and between the root/shoot ratio and RWC indicator despite the negligible negative correlation found with the Spearman rank correlation (**Table 1**). The WUE indicator and biomass penalty were relatively independent from root/shoot ratio and the NDVI indicator, hence they were likely situated in other areas of the drought resistance spectrum. Generally, accessions situated at the right-hand side of PC1 could be considered more drought-resistant, as a high value for the indicators based on NDVI, RWC, WUE and biomass penalty were all indicative of a poor resistance to drought. The different clusters derived from the cluster analyses separated very well along PC1 and PC2. Overall, accessions in clusters 1 and 2 seemed to respond less well to drought, while accessions in clusters 3, 4, and 5 are more drought resistant. Nonetheless, considerable variation in drought resistance was observed within clusters. When cultivated and wild accessions were colored separately in a PCA (**Supplementary Figure 9**), no obvious differences in drought resistance between wild and cultivated accessions could be observed. The cultivated accessions were distributed somewhat continuously along the entire PC1 axis.

### Relationship of drought resistance with local climate

**Figure 6:**
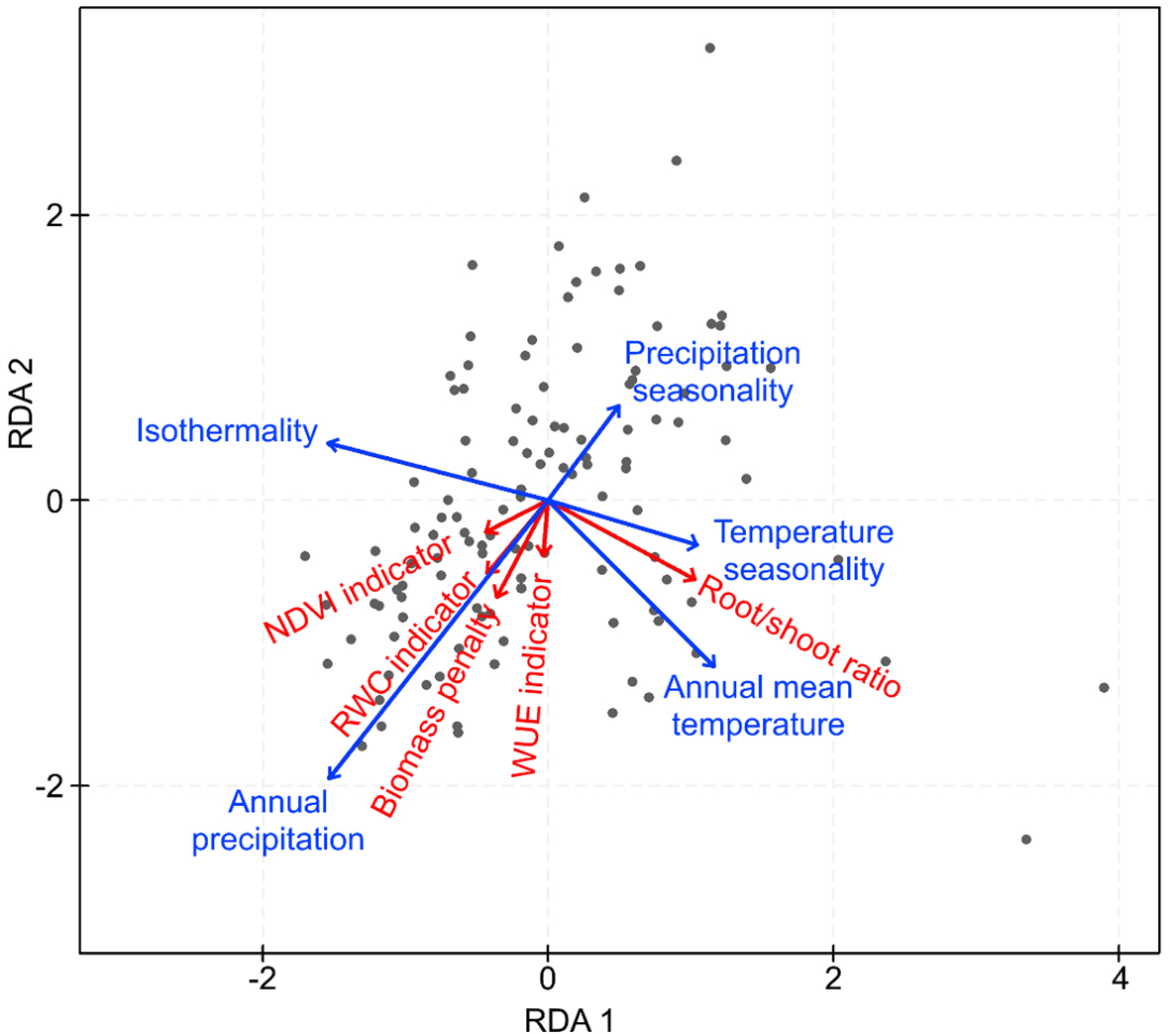
RDA analysis visualizing how climate conditions at the site of origin relate to the drought response indicator variables. The analysis was carried out with 118 accessions. Five climate variables (isothermality, precipitation seasonality, temperature seasonality, annual mean temperature, and annual precipitation) were included in the analysis, together with the five drought response indicator variables.

Only the first two axes of the RDA model were statistically significant (P < 0.001) and were retained (**Figure 6**). The included climatic variables (isothermality, precipitation seasonality, temperature seasonality, annual mean temperature, and annual precipitation) explained 14.2% (adjusted R²) of the variation in drought resistance among genotypes. Only three of the climatic variables (isothermality, annual mean temperature, and annual precipitation) were statistically significant (P < 0.001; P = 0.004; P < 0.001, respectively). Annual precipitation at the site of origin was significantly positively related to the indicators based on biomass penalty, RWC, and WUE (**Table 3**). Annual mean temperature was significantly positively related to the root/shoot ratio, while temperature seasonality decreased significantly with the indicator based on biomass penalty. Finally, a significant positive relation was observed between isothermality and biomass penalty, while a significant negative relation with root/shoot ratio was observed (**Table 3**).

**Table 3:** Pearson correlation coefficients for correlations between five drought response indicators and five climatic variables for 118 wild Phaseolinae genotypes.

	Annual mean temperature	Temperature seasonality	Annual precipitation	Precipitation seasonality	Isothermality
WUE	0.11	-0.08	0.20*	-0.08	0.01
RWC	-0.03	0.02	0.22*	-0.07	0.01
NDVI	-0.14	0.04	0.11	-0.15	0.02
Biomass penalty	0.11	-0.25**	0.40**	-0.14	0.19 *
Root/shoot ratio	0.36**	0.14	-0.17	0.02	-0.20**

Asterisks indicate p-values (*P < 0.05; **P < 0.01).

## Discussion

By using five drought resistance indicators to analyze the drought response of 151 Phaseolinae bean accessions, we were able to get a picture of the complexity of drought resistance across this agronomically important clade. The aim of studying multiple different indicators, on top of the biomass measurements that most drought resistance studies are limited to, was to gain a broader understanding of the effect of drought stress on plant morphology, physiology and molecular processes. Overall, the underlying cellular and molecular processes are all negatively affected by drought stress, which is demonstrated by the decreasing trend in biomass, WUE, RWC, and NDVI seen in virtually all accessions. However, we detected subtle differences between the indicators based on these variables. Most of the indicators were not correlated significantly to each other, confirming that their underlying molecular and cellular mechanisms can respond very differently to drought stress. The biomass penalty and WUE indicator were moderately correlated, which was to be expected as these indicators were both derived from the projected shoot area. The RWC and NDVI indicators were also moderately correlated, presumably because these indicators are both related to the photosynthesis process and derived from similar reflectance measurements. A strong correlation between NDVI and RWC has been observed in previous studies (e.g. Jiang et al., 2009). In remote sensing, NDVI has been shown to be a good proxy for biomass, and as such, it has also been used to analyze the response to drought in multiple studies. However, it has also been shown that this relation with biomass may disappear under severe drought conditions (Thapa et al., 2019). This might have been the case in our experiment, as plants were exposed to very low soil moisture contents. Hence, NDVI clearly captured a different drought response in our experiment.

Despite high intraspecific and within-subgenus variation, the cluster analysis was able to discern groups of accessions that show similar responses. In cluster 5, for instance, the drought response was mostly manifested through WUE, while all other drought resistance indicators revealed only a minor response. It has been argued that WUE might not provide much information about the competitive or yield advantage of one particular species over another because selection for improved WUE may actually restrict growth (Hubick et al., 1986; Blum, 2005, 2009). The trait has, however, been studied quite often because it can give an idea of the variation amongst genotypes in situations where water is limiting (Anyia and Herzog, 2004). In our study, accessions in cluster 3 were characterized by a high indicator value for both NDVI and RWC, suggesting that one of these two drought resistance indicators may be redundant. For RWC, despite its widespread use as an indicator for drought resistance, some studies have argued that the index may underestimate the actual water content of plants experiencing drought or high-salinity conditions, due to internal osmotic changes (Ievinsh, 2023). While absolute RWC measurements should thus be interpreted cautiously, our RWC-based indicator is only dependent on relative changes in the RWC (**Supplementary Figure 4**) and should thus not have been influenced by this effect. Our ability to differentiate various drought resistance clusters indicates that a high-throughput phenotyping platform enables a cost-efficient screening of drought resistances of highly diverse Phaseolinae genotypes, as further discussed below. Nonetheless, validation of our results by field trials remains necessary.

Root/shoot ratio was the drought resistance indicator that deviated most from all other indicators. Out of the five indicators, root/shoot ratio provided the lowest contribution to the first two principal components in our PCA analysis. The fact that the root/shoot ratio is also the indicator that was the least related to local precipitation data and seems to be more strongly related to annual temperature, suggests that the root/shoot ratio as measured here was not a sufficient indicator for drought resistance. Inherently, this metric ignores several characteristics of the root system that may influence drought resistance, such as its structure, effectiveness, and conductivity, as well as any phenological adaptations. Additionally, a low root biomass was here interpreted as characteristic for drought-sensitive plants. However, root growth can also be restricted in situations where the soil moisture is higher than required for optimal growth (Ran et al., 2023). This effect could have caused underestimation of the root/shoot ratio of highly drought-resistant plants that thrive in low soil moistures. The method for determining root biomass that was used here has its limitations as well. Since root biomass was estimated as the projected area of the roots in bottom-view, as imaged by an RGB camera, this indicator was based solely on the peripheral roots on the bottom of the pots. The projected root area measurements proved inaccurate during drought stress, as the dried-up soil formed clumps in the pots, pulling the roots away from the bottom of the pots. For this reason, we measured the root/shoot ratio before application of the drought stress, and we reasoned that it was therefore not a physiological response to drought stress but rather an indicator of potential drought resistance. Since the root/shoot ratio may respond differently for different species under drought stress (Zhou et al., 2018), further studies would benefit from a method to follow these changes during the drought stress period. In the past, electrical capacitance has been used for non-destructive root measuring. However, these measurements are strongly influenced by the soil moisture content, so making a fair comparison between plants under drought treatment and control plants is difficult (Cseresnyés et al., 2018). For now, no method exists for accurately determining root biomass under different soil conditions in a non-destructive manner.

While many studies have focused on screening for drought resistance in domesticated *Vigna* (e.g. Anyia and Herzog, 2004; Belko et al., 2012; Tsoata et al., 2015; Azimov et al., 2023) and *Phaseolus* species (Souza et al., 2003; Rosales et al., 2012; Mathobo et al., 2017; Mutari et al., 2023), far fewer studies have screened drought responses in the closely related wild relatives (but see Iseki et al., 2018). Cultivated species within the *Phaseolus* and *Vigna* genera face several environmental challenges that affect their global production, such as drought, extreme temperatures, and salinity (Beebe, 2012; Harouna et al., 2018). This is where wild relatives of crop plants may fill an important gap, as they often have greater adaptability to abiotic stresses and are expected to possess valuable genes for breeding (Acosta-Gallegos et al., 2007; Palmgren et al., 2014; Kouassi et al., 2020).

As far as we know, this is also the first study screening drought resistance across different genera and subgenera within the agronomically important Phaseolinae clade. One potential pitfall of screening drought resistance over such a wide diversity, is that different clades may have evolved different strategies to cope with drought stress. Although differences between subgenera were indeed observed, most variation was observed within subgenera. Even within species, considerable variation in drought resistance was observed. Accessions of *V. unguiculata*, for example, were found across four different drought resistance clusters, which confirms the findings of Iseki et al. (2018) for this species. On the other hand, accessions of *V. radiata* were remarkably similar in their drought response as all five of them clustered together in cluster 2. A large intraspecific diversity in drought resistance makes comparisons between different studies not using the same accessions difficult and shows the importance of using stable identifiers to characterize accessions and the exchange of information between seed banks across the world preserving and studying this material (Debouck, 2014; Nair et al., 2023). A few overall highly drought-resistant accessions worthy of mentioning are *V. (Vigna) racemosa* (NI 463), *V. (Ceratotropis) aconitifolia* (NI 482), *V. (Vigna) filicaulis* var. *filicaulis* (NI 1587), *V. (Ceratotropis) reflexo-pilosa* var. *reflexo-pilosa* (NI 1684), *V. (Plectotropis) vexillata* var. *ovata* (NI 1936), and *V. (Plectotropis) davyi* (NI 2024). Three of these varieties were phenotyped by Iseki et al. (2018) as well, although using different accessions. While they also found their accession of *V. vexillata* var. *ovata* to be among the most drought-resistant accessions, their accession of *V. reflexo-pilosa* var. *reflexo-pilosa* was found to be relatively drought-sensitive, and they found great variation in drought sensitivity in five different accessions of *V. (Ceratotropis) aconitifolia*. This again reinforces the idea of significant intraspecific diversity.

The redundancy analysis showed that all drought resistance indicators were to a varying extent related to climate at the original sampling locations of the wild accessions. A strong relationship was found between annual precipitation and biomass penalty, soil moisture at wilting (WUE), and soil moisture at leaf desiccation (RWC). Root/shoot ratio was related to annual mean temperature at the site of origin. These results validate the use of the high-throughput phenotyping platform for screening drought resistance across a wide diversity of Phaseolinae species and hint at stronger adaptation to drought for accessions and species that grow in dry regions. However, numerous other factors, such as vegetation structure, hydrogeography, altitude, and rhizobial and mycorrhizal interactions may influence the drought resistance of legumes (Cortés et al., 2013). In future studies, the influence of other factors could be explored. Mainly rhizobial and arbuscular mycorrhizal interactions, which have been shown to significantly alleviate drought stress effects in Fabaceae, should be considered (Rasaei et al., 2013; Tsoata et al., 2015; Oliveira et al., 2022).

No clear distinction could be found in drought resistance responses between cultivated and wild accessions. A PCA showed no separate grouping for wild and cultivated accessions. Moreover, cultivated accessions were found on both extreme ends of PC1 and in all five drought resistance clusters. This indicates that within cultivated Phaseolinae, considerable variation exists in drought resistance. This contrasts somewhat with the observations of Cortés et al. (2013), who found that the wild common bean (*Phaseolus vulgaris*) occupies more geographical regions with extensive drought stress than the cultivated accessions. Iseki et al. (2018) also observed that the most drought-resistant wild *Vigna* accessions performed better than the domesticated accessions that were cultivated in drought-prone areas. For subsequent studies, a more balanced number of accessions, both wild and cultivated, should be phenotyped for the important crop species, such as the common bean (*P. vulgaris*), Bambara groundnut (*V. subterranea*), and the mung bean (*V. radiata*). For the predicted climate changes, these accessions could be crucial in maintaining crop production.

High-throughput phenotyping platforms are increasingly used in agricultural settings as they come with several advantages. The main advantage is that large numbers of genotypes can be screened for abiotic stress responses, such as drought, in a time- and cost-efficient manner. We managed to screen the drought response for over 1500 plants from 151 accessions, with measurements taking place every 3 to 4 days. This would not have been possible with manual measurements. In addition, because the phenotyping platform is semi-automated, all measurements are relatively standardized and measurement error due to different observers was reduced. Using different types of cameras also allowed us to collect a huge amount of data variables, of which only a subset was used in the present analysis. With this study, we have shown that high-throughput phenotyping can also aid screening of wild genotypes, which are generally much more variable in terms of plant size and growth habit (Popoola et al., 2015) as compared to varieties within a single species.

There were also some limitations to the high-throughput phenotyping platform. All accessions were subjected to the same drought treatment, meaning that accessions with high water uptake experienced more severe levels of drought stress earlier in the experiment. This is reflected in the variation in the number of days it took for each plant to reach the threshold soil moisture value of 20%. While the large majority of plants reached this soil moisture after 29, 33, or 36 DAS, several plants showed a significantly slower or faster soil moisture decrease. To alleviate this bias, the indicator based on WUE was included, since WUE accounts for plant size and thus partially for water uptake. To further reduce this bias, future studies could benefit from a method to equalize the drought treatment between different accessions, such as partial recycling of the transpired water, in a high-throughput manner. If the experimental setup allows for it, applying different levels of drought severity depending on the accessions’ physiology could also allow a more nuanced comparison. Another limitation was that the drought experiment was run for a limited amount of time, as when plants grew too large, they had to be tied up to sticks regularly, since many of the Phaseolinae accessions are climbers. This may have reduced measurement accuracy at some point. Due to this time limit, the type of stress provided was also quite severe, which may not be representative of all natural situations. Plant response to a given water deficit is for example strongly dependent on the previous occurrence and intensity of other drought stress events and the presence of other stresses (Seleiman et al., 2021). A relevant example of a parameter evaluated here is plant wilting, which is dependent not only on soil moisture, but also on vapor pressure deficit, light exposure, root conductivity, and other factors (Schultz and Matthews, 1997). A logical next step would be to run experiments with different types of water deficit treatments, such as a prolonged exposure to drought, and in interaction with variation of other environmental conditions, such as variation in temperature. This would however increase the number of biological replicates required, which would necessitate an even higher throughput. Finally, tying the information obtained through our high-throughput phenotyping experiment with anatomical measurements (e.g., stomatal density and size), phenology (e.g. flowering and pod production), and functional traits (stomatal conductance and transpiration rate) related to stress resistance will help to elucidate the role of each component in the adaptation to fluctuations in water deficit (Correia et al., 2022).

In conclusion, our study provides an explorative approach to simultaneously characterize the changes in selected morphological, physiological, and molecular plant traits under drought stress. Our results demonstrate that drought resistance in Phaseolinae beans and, by extension, other crop plants, can manifest itself in multiple different ways, and cannot be simply characterized by quantifying the biomass. The indicators soil moisture at wilting derived from WUE curves, soil moisture at leaf desiccation derived from RWC curves, and soil moisture at chlorosis derived from NDVI curves, together with a biomass penalty, provide a broad and nuanced vision on the complexities of a plant’s drought response. We derived these indicators for 151 accessions of both wild and cultivated *Vigna* and *Phaseolus* bean species. Our study thus revealed accessions that may be of interest in breeding programs. We demonstrated the presence of large within-subgenus differences in the capacity to resist drought stress, which could be exploited in breeding. Lastly, we compared drought resistance of the accessions with local climate data of their collection sites and found strong relationships with precipitation data, confirming that the most drought-resistant Phaseolinae accessions mainly grow in arid climates. In order to maintain or increase our current global bean production in times of increasing drought disasters, further studies should expand upon this knowledge gained from phenotypic traits. Both genomic information and studies on the rhizobial and mycorrhizal interactions could provide invaluable insights.

## Data Availability

The datasets presented in this study can be found in online repositories. The names of the repository/repositories and accession number(s) can be found in the article/**Supplementary Material**. The datasets generated for this study can be found on Zenodo at https://zenodo.org/records/11179671.

## References

[B1] Acosta-Gallegos J. Kelly J. D. Gepts P. (2007). Prebreeding in common bean and use of genetic diversity from wild germplasm. Crop Sci. 47, S–44-S59. doi: 10.2135/cropsci2007.04.0008IPBS

[B2] Anyia A. O. Herzog H. (2004). Water-use efficiency, leaf area and leaf gas exchange of cowpeas under mid-season drought. Eur. J. Agron. 20, 327–339. doi: 10.1016/S1161-0301(03)00038-8

[B3] Azimov A. Shavkiev J. Saidjanov S. Ziyaev Z. Valiyev L. (2023). Mung Bean (Vigna radiata L.) genotypes assessment for drought tolerance in Uzbekistan. J. Wildlife Biodiversity 8, 65–75. doi: 10.5281/zenodo.10171284

[B4] Bandurska H. (2022). Drought stress responses: coping strategy and resistance. Plants (Basel) 11, 922. doi: 10.3390/plants11070922 35406902 PMC9002871

[B5] Beebe S. (2012). “Common bean breeding in the tropics,” in Plant Breeding Reviews 36. Ed. Janick J. (Wiley-Blackwell, Oxford), 357–426. doi: 10.1002/9781118358566.ch5

[B6] Belko N. Zaman-Allah M. Cisse N. Diop N. N. Zombre G. Ehlers J. D.. (2012). Lower soil moisture threshold for transpiration decline under water deficit correlates with lower canopy conductance and higher transpiration efficiency in drought-tolerant cowpea. Funct. Plant Biol. 39, 306–322. doi: 10.1071/FP11282 32480783

[B7] Bell G. E. Howell B. M. Johnson G. V. Raun W. R. Solie J. B. Stone M. L. (2004). Optical sensing of turfgrass chlorophyll content and tissue nitrogen. HortScience 39, 1130–1132. doi: 10.21273/HORTSCI.39.5.1130

[B8] Blum A. (2005). Drought resistance, water-use efficiency, and yield potential-are they compatible, dissonant, or mutually exclusive? Aust. J. Agric. Res. 56, 1159–1168. doi: 10.1071/AR05069

[B9] Blum A. (2009). Effective use of water (EUW) and not water-use efficiency (WUE) is the target of crop yield improvement under drought stress. Field Crops Res. 112, 119–123. doi: 10.1016/j.fcr.2009.03.009

[B10] Borrell A. K. Hammer G. L. Henzell R. G. (2000). Does maintaining green leaf area in sorghum improve yield under drought? II. Dry matter production and yield. Crop Sci. 40, 1037–1048. doi: 10.2135/cropsci2000.4041037x

[B11] Briggs L. J. Shantz H. L. (1913). The water requirement of plants (Washington, D.C: U.S. Government Printing Office).

[B12] Carrão H. Naumann G. Barbosa P. (2016). Mapping global patterns of drought risk: An empirical framework based on sub-national estimates of hazard, exposure and vulnerability. Global Environ. Change 39, 108–124. doi: 10.1016/j.gloenvcha.2016.04.012

[B13] Condorelli G. E. Maccaferri M. Newcomb M. Andrade-Sanchez P. White J. W. French A. N.. (2018). Comparative aerial and ground based high throughput phenotyping for the genetic dissection of NDVI as a proxy for drought adaptive traits in durum wheat. Front. Plant Sci. 9. doi: 10.3389/fpls.2018.00893 PMC6028805 29997645

[B14] Correia P. M. P. Westergaard J. C. da Silva A. B. Roitsch T. Carmo-Silva E. da Silva J. M. (2022). High-throughput phenotyping of physiological traits for wheat resilience to high temperature and drought stress. J. Exp. Bot. 73, 5235–5251. doi: 10.1093/jxb/erac160 35446418 PMC9440435

[B15] Cortés A. J. Monserrate F. A. Ramírez-Villegas J. Madriñán S. Blair M. W. (2013). Drought tolerance in wild plant populations: the case of common beans (*Phaseolus vulgaris* L.). PLoS One 8, e62898. doi: 10.1371/journal.pone.0062898 23658783 PMC3643911

[B16] Cseresnyés I. Szitár K. Rajkai K. Füzy A. Mikó P. Kovács R.. (2018). Application of electrical capacitance method for prediction of plant root mass and activity in field-grown crops. Front. Plant Sci. 9. doi: 10.3389/fpls.2018.00093 PMC5799269 29449861

[B17] Daryanto S. Wang L. Jacinthe P.-A. (2016). Global synthesis of drought effects on maize and wheat production. PLoS One 11, e0156362. doi: 10.1371/journal.pone.0156362 27223810 PMC4880198

[B18] Debouck D. G. (2014). Conservation of Phaseolus beans genetic resources: A strategy (Rome: Global Crop Diversity Trust).

[B19] de Carvalho M. H. C. (2008). Drought stress and reactive oxygen species: production, scavenging and signaling. Plant Signaling Behav. 3, 156–165. doi: 10.4161/psb.3.3.5536 PMC2634109 19513210

[B20] Dempewolf H. Eastwood R. J. Guarino L. Khoury C. K. Müller J. V. Toll J. (2014). Adapting agriculture to climate change: A global initiative to collect, conserve, and use crop wild relatives. Agroecology Sustain. Food Syst. 38, 369–377. doi: 10.1080/21683565.2013.870629

[B21] Elzhov T. V. Mullen K. M. Spiess A. Bolker B. (2023). _minpack.lm: R Interface to the Levenberg-Marquardt Nonlinear Least-Squares Algorithm Found in MINPACK, Plus Support for Bounds_ (R package version 1.2-4). Available at: https://CRAN.R-project.org/package=minpack.lm.

[B22] Fick S. E. Hijmans R. J. (2017). WorldClim 2: new 1-km spatial resolution climate surfaces for global land areas. Int. J. Climatology. 37, 4302–4315. doi: 10.1002/joc.5086

[B23] Fleischer W. E. (1935). The relation between chlorophyll content and rate of photosynthesis. J. Gen. Physiol. 18, 573–597. doi: 10.1085/jgp.18.4.573 19872868 PMC2141364

[B24] Food and Agriculture Organization of the United Nations (2020). Data from: Crops and livestock products (Rome: FAOSTAT Statistical Database). Available at: https://www.fao.org/faostat/en/#data/QCL.

[B25] Galili T. (2015). dendextend: an R package for visualizing, adjusting and comparing trees of hierarchical clustering. Bioinformatics 31, 3718–3720. doi: 10.1093/bioinformatics/btv428 26209431 PMC4817050

[B26] González-Espíndola L.Á. Pedroza-Sandoval A. Trejo-Calzada R. Jacobo-Salcedo M. D. R. de los Santos G. G. Quezada-Rivera J. J. (2024). Relative water content, chlorophyll index, and photosynthetic pigments on *lotus corniculatus* L. @ in response to water deficit. Plants 13, 961. doi: 10.3390/plants13070961 38611490 PMC11013262

[B27] Haider Z. Khan A. S. Zia S. (2012). Correlation and path coefficient analysis of yield components in rice (*Oryza sativa* L.) under simulated drought stress condition. American-Eurasian J. Agric. Environ. Sci. 12, 100–104.

[B28] Harouna D. V. Venkataramana P. B. Ndakidemi P. A. Matemu A. O. (2018). Under-exploited wild Vigna species potentials in human and animal nutrition: A review. Global Food Secur. 18, 1–11. doi: 10.1016/j.gfs.2018.06.002

[B29] Hijmans R. J. Guarino L. Cruz M. Rojas E. (2001). Computer tools for spatial analysis of plant genetic resources data: 1. DIVA-GIS. Plant Genet. Resour. Newslett. 127, 15–19.

[B30] Hubick K. T. Farquhar G. D. Shorter R. (1986). Correlation between water-use efficiency and carbon isotope discrimination in diverse peanut (*Arachis*) germplasm. Aust. J. Plant Physiol. 13, 803–816. doi: 10.1071/PP9860803

[B31] Ievinsh G. (2023). Water content of plant tissues: so simple that almost forgotten? Plants 12, 1238. doi: 10.3390/plants12061238 36986926 PMC10058729

[B32] Ikram S. Bhattarai S. Walsh K. B. (2024). Screening new mungbean varieties for terminal drought tolerance. Agriculture 14, 1328. doi: 10.3390/agriculture14081328

[B33] IPCC. (2021). “Summary for policymakers,” in Climate Change 2021: The Physical Science Basis. Eds. Masson-Delmotte M. I. Zhai P. Pirani A. (Cambridge University Press, Cambridge, New York), 3–32.

[B34] Iseki K. Takahashi Y. Muto C. Naito K. Tomooka N. (2018). Diversity of drought tolerance in the genus vigna. Front. Plant Sci. 9. doi: 10.3389/fpls.2018.00729 PMC6014140 29963062

[B35] Javornik T. Carović-Stanko K. Gunjača J. Vidak M. Lazarević B. (2023). Monitoring drought stress in common bean using chlorophyll fluorescence and multispectral imaging. Plants 12, 1386. doi: 10.3390/plants12061386 36987074 PMC10059887

[B36] Jiang Y. Liu H. Cline V. (2009). Correlations of leaf relative water content, canopy temperature, and spectral reflectance in perennial ryegrass under water deficit conditions. HortScience 44, 459–462. doi: 10.21273/HORTSCI.44.2.459

[B37] Kassambara A. Mundt F. (2020). Package ‘factoextra’: Extract and Visualize the Results of Multivariate Data Analyses (R package version 1.0.7). Available online at: https://CRAN.Rproject.org/package=factoextra (Accessed December 2023).

[B38] Kim D. M. Zhang H. Zhou H. Du T. Wu Q. Mockler T. C.. (2015). Highly sensitive image-derived indices of water-stressed plants using hyperspectral imaging in SWIR and histogram analysis. Sci. Rep. 5, 15919. doi: 10.1038/srep15919 26531782 PMC4632122

[B39] Kouassi A. B. Kouassi K. B. A. Sylla Z. Plazas M. Fonseka R. M. Kouassi A.. (2020). Genetic parameters of drought tolerance for agromorphological traits in eggplant, wild relatives, and interspecific hybrids. Crop Sci. 61, 55–68. doi: 10.1002/csc2.20250

[B40] Liao X. Meyer M. C. (2019). cgam: an R package for the constrained generalized additive model. J. Stat. Software 89, 1–24. doi: 10.18637/jss.v089.i05

[B41] Ma H. Mo L. Crowther T. W. Maynard D. S. van den Hoogen J. Stocker B. D.. (2021). The global distribution and environmental drivers of aboveground versus belowground plant biomass. Nat. Ecol. Evol. 5, 1110–1122. doi: 10.1038/s41559-021-01485-1 34168336

[B42] Maréchal R. Mascherpa J.-M. Stainier F. (1978). Étude taxonomique d'un groupe complexe d'espèces des genres Phaseolus et Vigna (Papilionaceae) sur la base de données morphologiques et polliniques, traitées par l'analyse informatique (Genève: Conservatoire et jardin botaniques).

[B43] Mathobo R. Marais D. Steyn J. M. (2017). The effect of drought stress on yield, leaf gaseous exchange and chlorophyll fluorescence of dry beans (*Phaseolus vulgaris* L.). Agric. Water Manage. 180, 118–125. doi: 10.1016/j.agwat.2016.11.005

[B44] Miguez F. (2019). Package ‘nlraa’: Nonlinear Regression for Agricultural Applications (R package version 1.9.3). Available online at: https://CRAN.R-project.org/package=nlraa (Accessed December 2023).

[B45] Mutari B. Sibiya J. Shayanowako A. Chidzanga C. Matova P. M. Gasura E. (2023). Genome-wide association mapping for component traits of drought tolerance in dry beans (*Phaseolus vulgaris* L.). PLoS One 18, e0278500. doi: 10.1371/journal.pone.0278500 37200295 PMC10194967

[B46] MVTec Software GmbH. (2021). HALCON – The Power of Machine Vision. Available online at: https://www.mvtec.com/products/halcon (Accessed May 2021).

[B47] Nair R. M. Pujar M. Cockel C. Scheldeman X. Vandelook F. van Zonneveld M.. (2023). Global strategy for the conservation and use of *Vigna.* (Rome: Global Crop Diversity Trust).

[B48] Oksanen J. Simpson G. L. Blanchet F. G. Kindt R. Legendre P. Minchin P. R.. (2022). Package ‘vegan’: Community Ecology Package (R package version 2.6-4). Available online at: https://CRAN.R-project.org/package=vegan (Accessed December 2023).

[B49] Oliveira T. C. Cabral J. S. R. Santana L. R. Tavares G. G. Santos L. D. S. Paim T. P.. (2022). The arbuscular mycorrhizal fungus *Rhizophagus clarus* improves physiological tolerance to drought stress in soybean plants. Sci. Rep. 12, 9044. doi: 10.1038/s41598-022-13059-7 35641544 PMC9156723

[B50] Palmgren M. G. Edenbrandt A. K. Vedel S. E. Andersen M. M. Landes X. Østerberg J. T.. (2014). Are we ready for back-to-nature crop breeding? Trends Plant Sci. 20, 155–164. doi: 10.1016/j.tplants.2014.11.003 25529373

[B51] Popoola J. O. Aremu B. R. Daramola F. Y. Ejoh A. S. Adegbite A. E. (2015). Morphometric analysis of some species in the genus *Vigna* (L.) Walp: implication for utilization for genetic improvement. J. Biol. Sci. 15, 156–166. doi: 10.3923/jbs.2015.156.166

[B52] Qi Y. Wei W. Chen C. Chen L. (2019). Plant root-shoot biomass allocation over diverse biomes: A global synthesis. Global Ecol. Conserv. 18, e00606. doi: 10.1016/j.gecco.2019.e00606

[B53] Ran X. Qiao S. Zhang Y. Gao X. Du Y. Liu B.. (2023). Study on the causes of growth differences in three conifers after the rainy season in the Xiong’an New Area. Front. Plant Sci. 14. doi: 10.3389/fpls.2023.1176142 PMC10352786 37469775

[B54] Rasaei B. Ghobadi M. E. Ghobadi M. Najaphy A. (2013). Reducing effects of drought stress by application of humic acid, Mycorrhiza and Rhizobium on chickpea. Int. J. Agric. Crop Sci. 5, 1775–1778. doi: 10.5555/20133304333

[B55] R Core Team. (2022). R: A language and environment for statistical computing (Vienna: R Foundation for Statistical Computing). Available at: https://www.R-project.org/.

[B56] Reddy S. S. Singh G. M. Kumar U. Bhati P. Vishwakarma M. Navathe S.. (2024). Spatio-temporal evaluation of drought adaptation in wheat revealed NDVI and MTSI as powerful tools for selecting tolerant genotypes. Field Crops Res. 311, 109367. doi: 10.1016/j.fcr.2024.109367

[B57] Rosales M. A. Ocampo E. Rodríguez-Valentín R. Olvera-Carrillo Y. Acosta-Gallegos J. Covarrubias A. A. (2012). Physiological analysis of common bean (*Phaseolus vulgaris* L.) cultivars uncovers characteristics related to terminal drought resistance. Plant Physiol. Biochem. 56, 24–34. doi: 10.1016/j.plaphy.2012.04.007 22579941

[B58] Rouse J. W. Haas R. H. Schell J. A. Deering D. W. (1974). Monitoring vegetation systems in the Great Plains with ERTS. Third Earth Resour. Technol. Satellite-1 Symposium 1, 309–317.

[B59] RStudio Team. (2022). RStudio: Integrated Development for R. Boston (RStudio, PBC). Available at: http://www.rstudio.com/.

[B60] SAS Institute Inc. (2019). SAS® Studio 5.2 (Cary: SAS Institute Inc). Available at: https://www.sas.com/en_be/software/studio.html.

[B61] Schonfeld M. A. Johnson R. C. Carver B. F. Mornhinweg D. W. (1988). Water Relations in winter wheat as drought resistance indicators. Crop Sci. 28, 526–531. doi: 10.2135/cropsci1988.0011183X002800030021x

[B62] Schultz H. R. Matthews M. A. (1997). High vapour pressure deficit exacerbates xylem cavitation and photoinhibition in shade-grown *Piper auritum* H.B. & K. during prolonged sunflecks. Oecologia 110, 312–319. doi: 10.1007/s004420050164 28307219

[B63] Seleiman M. F. Al-Suhaibani N. Ali N. Akmal M. Alotaibi M. Refay Y.. (2021). Drought stress impacts on plants and different approaches to alleviate its adverse effects. Plants 10, 259. doi: 10.3390/plants10020259 33525688 PMC7911879

[B64] Shantz H. L. (1927). Drought resistance and soil moisture. Ecology 8, 145–157. doi: 10.2307/1928954

[B65] Siddique M. R. B. Hamid A. Islam M. S. (2000). Drought stress effects on water relations of wheat. Botanical Bull. Academia Sin. 41, 35–39.

[B66] Sofi P. A. Djanaguiraman M. Siddique K. H. M. Prasad P. V. V. (2018). Reproductive fitness in common bean (*Phaseolus vulgaris* L.) under drought stress is associated with root length and volume. Indian J. Plant Physiol. 23, 796–809. doi: 10.1007/s40502-018-0429-x

[B67] Souza G. M. de Tarso Aidar S. Giaveno C. D. de Oliveira R. F. (2003). Drought stability in different common bean (*Phaseolus vulgaris* L.) genotypes. Crop Breed. Appl. Biotechnol. 3, 203–208. doi: 10.12702/1984-7033.v03n03a04

[B68] Thapa S. Rudd J. C. Xue Q. Bhandari M. Reddy S. K. Jessup K. E.. (2019). Use of NDVI for characterizing winter wheat response to water stress in a semi-arid environment. J. Crop Improvement 33, 633–648. doi: 10.1080/15427528.2019.1648348

[B69] Trapp J. J. Urrea C. A. Zhou J. Khot L. R. Sankaran S. Miklas P. N. (2016). Selective phenotyping traits related to multiple stress and drought response in dry bean. Crop Sci. 56, 1460–1472. doi: 10.2135/cropsci2015.05.0281

[B70] Tsoata E. Njock S. R. Youmbi E. Nwaga D. (2015). Early effects of water stress on some biochemical and mineral parameters of mycorrhizal *Vigna subterranea* (L.) Verdc. (Fabaceae) cultivated in Cameroon. Int. J. Agron. Agric. Res. 7, 21–35.

[B71] Verheyen J. Dhondt S. Abbeloos R. Eeckhout J. Janssens S. Leyns F.. (2024). High-throughput phenotyping reveals multiple drought responses of wild and cultivated Phaseolinae beans. bioRxiv. doi: 10.1101/2024.02.09.579595v1 PMC11466915 39399541

[B72] VIB. (2021). PIPPA. Version 0.14.20. Available online at: https://pippa.psb.ugent.be/ (Accessed October 17th, 2021).

[B73] Vu V. Q. (2011). Package ‘ggbiplot’: A Grammar of Graphics Implementation of Biplots (R package version 0.6.2). Available online at: https://CRAN.R-project.org/package=ggbiplot (Accessed December 2023).

[B74] WFO. (2022a). Phaseolus. Available online at: http://www.worldfloraonline.org/taxon/wfo-4000029154 (Accessed May 2022).

[B75] WFO. (2022b). Vigna. Available online at: http://www.worldfloraonline.org/taxon/wfo-4000040261 (Accessed May 2022).

[B76] Wickham H. (2016). ggplot2: Elegant Graphics for Data Analysis (New York: Springer-Verlag).

[B77] Zhou G. Zhou X. Nie Y. Bai S. H. Zhou L. Shao J.. (2018). Drought-induced changes in root biomass largely result from altered root morphological traits: Evidence from a synthesis of global field trials. Plant Cell Environ. 41, 2589–2599. doi: 10.1111/pce.13356 29879755

