# Supplementary material for: High-throughput phenotyping reveals multiple drought responses of wild and cultivated Phaseolinae beans
Source: Front Plant Sci. 2024 Sep 27;15:1385985. doi: 10.3389/fpls.2024.1385985 (PMC11466915; doi:10.3389/fpls.2024.1385985)

**Supplementary data**

*Supplementary Table 1.* See supplementary Excel file.

*Supplementary Table 2.* Profile of the soil used in the experiment.

| **Soil ‘Potgrond voor Zaaien en Stekken’ (Saniflor)** | |
| --- | --- |
| Parameter | Value in kg/1000 kg |
| Dry matter | 327 |
| Organic matter (SOM, loss on ignition) | 276 |
| Organic carbon (SOC, 0.58 × SOM) | 160.1 |
| Total nitrogen (N) | 4.11 |
| Mineral nitrogen (ammonium-N + nitrate-N) | 0.70 |
| Phosphorus (P_2_O_5_) | 0.41 |
| Potassium (K_2_O) | 0.230 |
| Magnesium (MgO) | 1.25 |
| Calcium (CaO) | 11.5 |
| Sodium (Na_2_O) | 0.107 |
| Parameter | Value in g/1000 kg |
| Arsenic (As) | < 3.27 |
| Cadmium (Cd) | < 0.164 |
| Chromium (Cr) | < 1.64 |
| Copper (Cu) | 3.40 |
| Mercury (Hg) | < 0.0327 |
| Nickel (Ni) | < 1.64 |
| Lead (Pb) | < 6.5 |
| Zinc (Zn) | 4.8 |
| Parameter | Value |
| pH (H_2_O) | 6.6 |
| Bulk density | 0.301 kg/L |
| Electrical conductance (EC) | 230 µS/cm 25°C |
| Cation exchange capacity (CEC) | 66 cmol_C_/kg |
| C/N ratio | 38.9 |
| **3 g/L Osmocote® Exact Mini 3-4M (Everris)** | |
| Parameter | Percentage |
| Total nitrogen (N) | 16% |
| Nitrate nitrogen (N-NO_3_) | 7% |
| Ammoniacal nitrogen (N-NH_4_) | 9% |
| Phosphorus pentoxide (P_2_O_5_) | 9% |
| Water soluble P_2_O_5_ | 6.5% |
| Potassium oxide (K_2_O) | 12% |
| Water soluble K_2_O | 12% |
| Magnesium oxide (MgO) | 2% |
| Water soluble MgO | 1.3% |
| Iron (Fe) | 0.45% |
| Fe chelated by EDTA | 0.09% |
| Manganese (Mn) | 0.065% |
| Boron (B) | 0.024% |
| Water soluble B | 0.022% |
| Copper (Cu) | 0.06% |
| Water soluble Cu | 0.047% |
| Molybdenum (Mo) | 0.024% |
| Water soluble Mo | 0.024% |
| Zinc (Zn) | 0.028% |
| Water soluble Zn | 0.019% |

*Supplementary Table 3.* The proportion of within-subgenus variability and between-subgenus variability to the total variability in drought response indicator values based a mixed model analysis with subgenus as random effect. In addition, heterogeneous within-subject variability was modelled by estimating a different residual variance for each subgenus.

| (Sub)genus | Within variability | Total variability | Within variability (%) |
| --- | --- | --- | --- |
| WUE indicator values | | | |
| *V. (Ceratotropis)* | 0.0025 | 0.0030 | 83% |
| *Condylostylis* | 0.0018 | 0.0024 | 78% |
| *V. (Haydonia)* | 0.0006 | 0.0011 | 54% |
| *V. (Lasiospron)* | 0.0026 | 0.0031 | 83% |
| *P. (Phaseolus)* | 0.0020 | 0.0025 | 80% |
| *V. (Plectrotropis)* | 0.0023 | 0.0028 | 81% |
| *Sigmoidotropis* | 0.0001 | 0.0007 | 21% |
| *V. (Vigna)* | 0.0028 | 0.0033 | 85% |
| RWC indicator values | | | |
| *V. (Ceratotropis)* | 0.0042 | 0.0083 | 50% |
| *Condylostylis* | 0.0073 | 0.0114 | 64% |
| *V. (Haydonia)* | 0.0082 | 0.0123 | 66% |
| *V. (Lasiospron)* | 0.0009 | 0.0050 | 17% |
| *P. (Phaseolus)* | 0.0050 | 0.0091 | 55% |
| *V. (Plectrotropis)* | 0.0046 | 0.0087 | 52% |
| *Sigmoidotropis* | 0.0070 | 0.0111 | 63% |
| *V. (Vigna)* | 0.0061 | 0.0102 | 59% |
| NDVI indicator values | | | |
| *V. (Ceratotropis)* | 0.0063 | 0.0068 | 92% |
| *Condylostylis* | 0.0197 | 0.0203 | 97% |
| *V. (Haydonia)* | 0.0046 | 0.0052 | 89% |
| *V. (Lasiospron)* | 0.0014 | 0.0019 | 71% |
| *P. (Phaseolus)* | 0.0114 | 0.0119 | 95% |
| *V. (Plectrotropis)* | 0.0033 | 0.0039 | 86% |
| *Sigmoidotropis* | 0.0160 | 0.0165 | 97% |
| *V. (Vigna)* | 0.0084 | 0.0090 | 94% |
| Biomass indicator values | | | |
| *V. (Ceratotropis)* | 0.039 | 0.0491 | 80% |
| *Condylostylis* | 0.0396 | 0.0495 | 80% |
| *V. (Haydonia)* | 0.0373 | 0.0473 | 79% |
| *V. (Lasiospron)* | 0.0037 | 0.0137 | 27% |
| *P. (Phaseolus)* | 0.0371 | 0.0470 | 79% |
| *V. (Plectrotropis)* | 0.0933 | 0.1033 | 90% |
| *Sigmoidotropis* | 0.0533 | 0.0633 | 84% |
| *V. (Vigna)* | 0.0211 | 0.0311 | 68% |
| Root/shoot ratio | | | |
| *V. (Ceratotropis)* | 0.0142 | 0.0150 | 94% |
| *Condylostylis* | 0.0007 | 0.0017 | 44% |
| *V. (Haydonia)* | 0.0016 | 0.0026 | 64% |
| *V. (Lasiospron)* | 0.0005 | 0.0014 | 33% |
| *P. (Phaseolus)* | 0.0012 | 0.0021 | 56% |
| *V. (Plectrotropis)* | 0.0008 | 0.0017 | 46% |
| *Sigmoidotropis* | 0.0009 | 0.0019 | 50% |
| *V. (Vigna)* | 0.0048 | 0.0057 | 84% |

*Supplementary Figure 1.* Setup of the experiment. (A) Pots were arranged on flooding tables in a completely randomized design. (B) For watering, the flooding tables were completely flooded, so that water could enter the pots through holes in the bottom. Afterwards, the water was drained from the tables, so that all excess water also drained from the pots. (C) The high-throughput phenotyping system using conveyor belts.


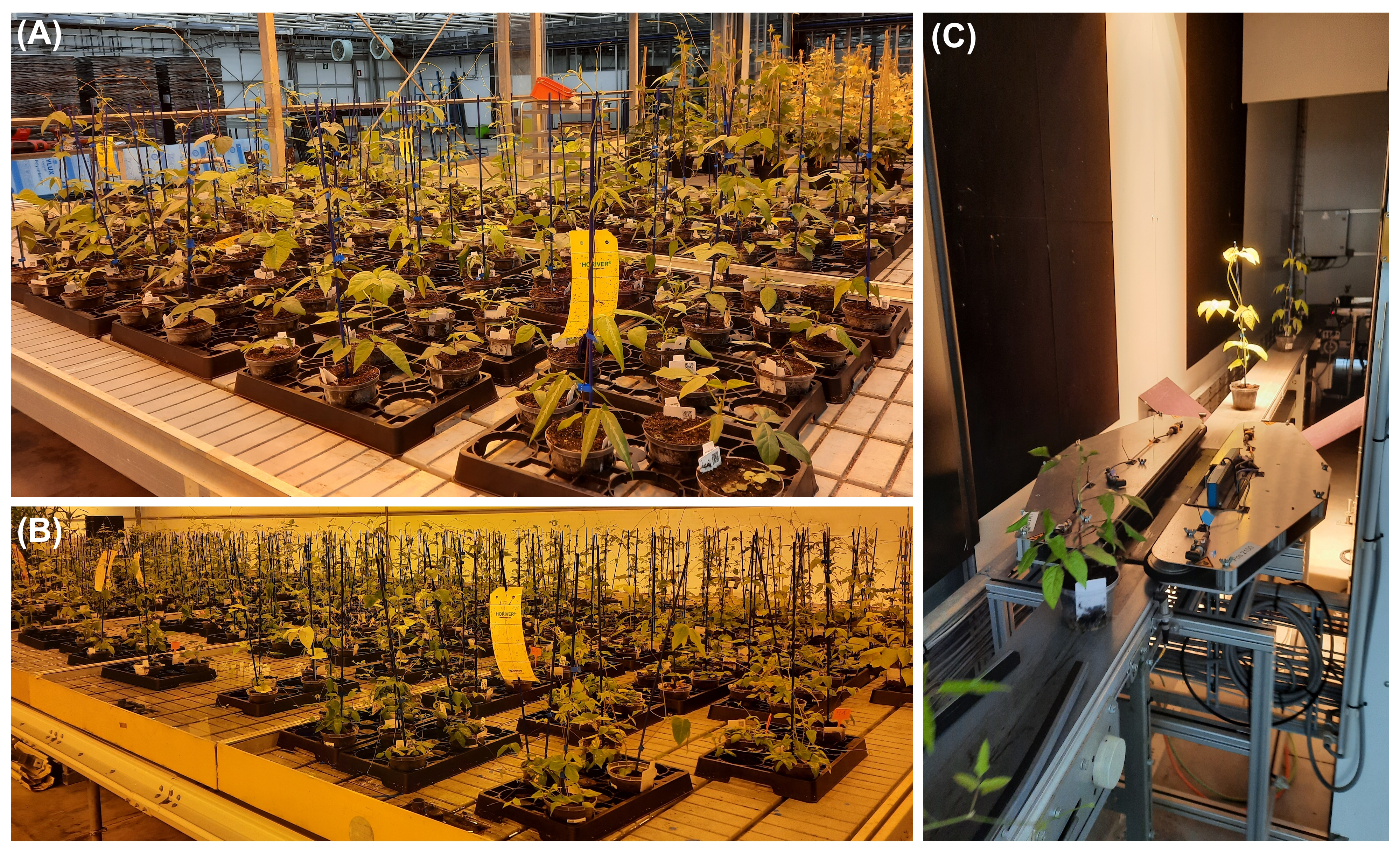


*Supplementary Figure 2.* Greenhouse temperature and relative humidity during the experiment.


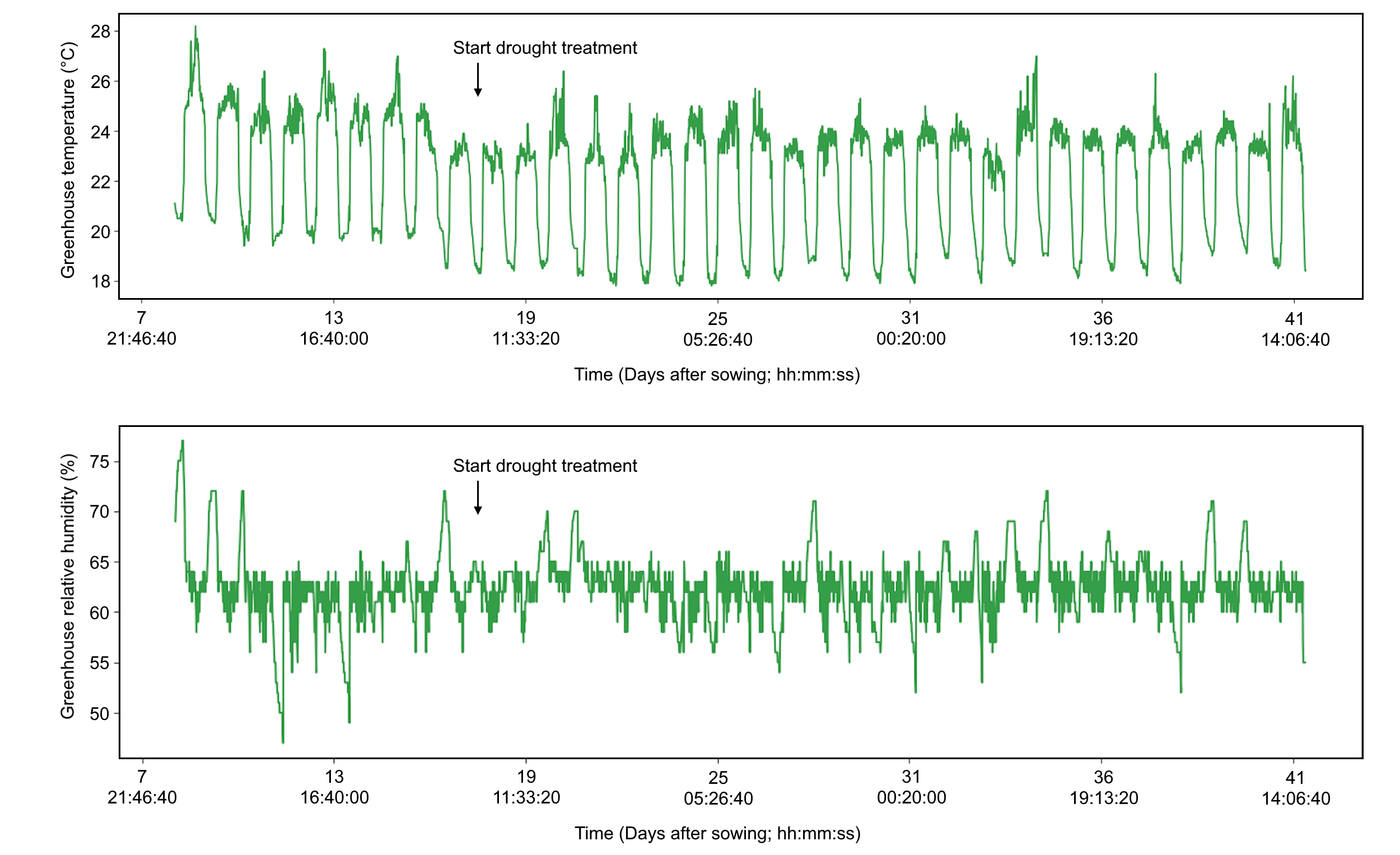


*Supplementary Figure 3.* The linear relationship between the projected front area and the aboveground biomass was determined in a preliminary experiment based on 20 accessions with 8 to 10 plants for each accession using the *reg* procedure in SAS Studio version 5.2 (SAS Institute Inc., Cary, NC, USA).


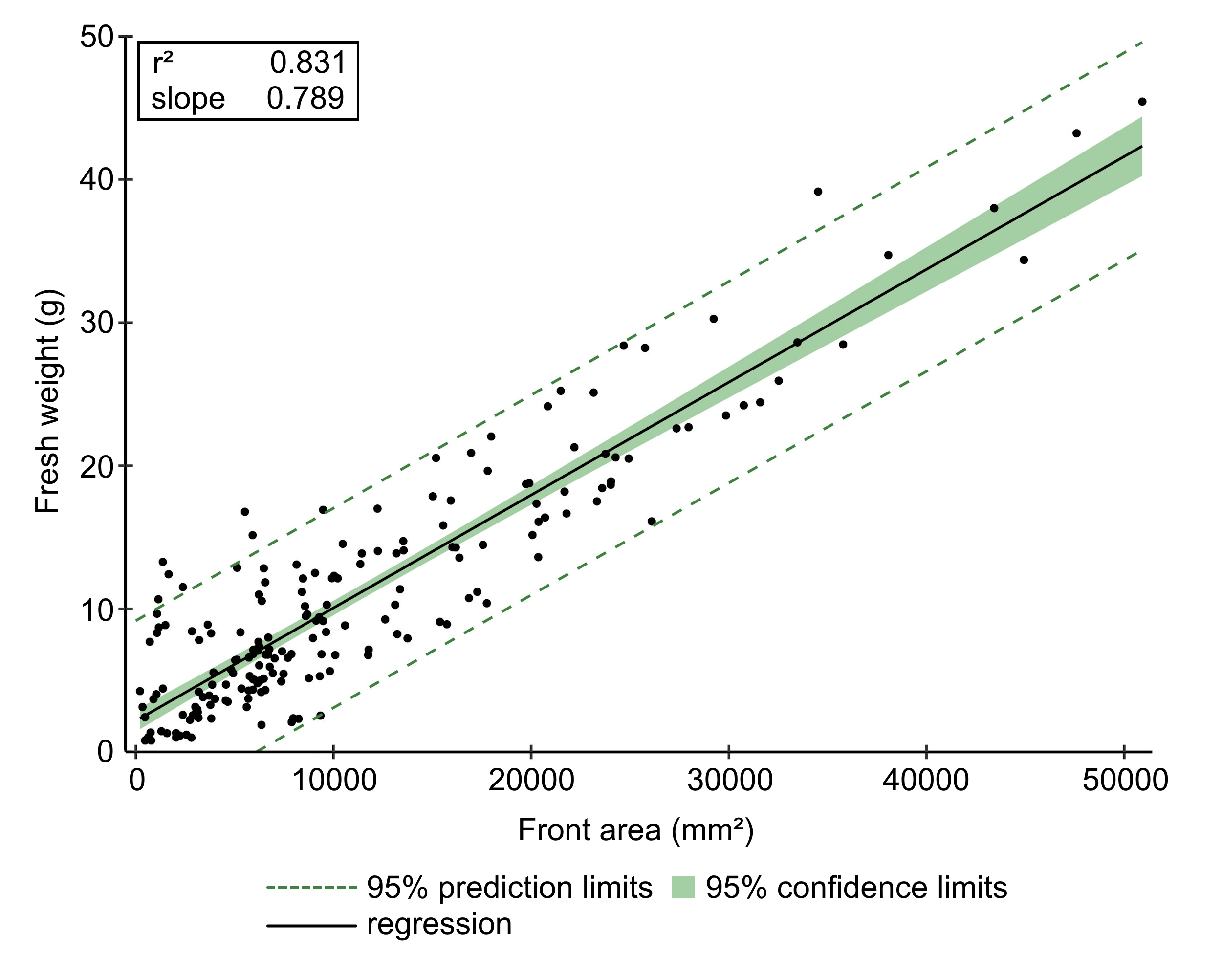


*Supplementary Figure 4*. Explanatory graphs for each of the five indicators. For each panel, the left figure gives an example of a drought resistant accession, and the right figure gives an example of a drought sensitive accession, based on the indicator on that panel. (A) Projected side area of drought and control groups as an estimator for biomass. Biomass penalty was calculated as the difference of the means (indicated by crosses), divided by the mean of the control group. (B) WUE plotted against soil moisture and fitted with a CGAM model curve. Colors indicate individual plants. The intercept of the curve with the x-axis is the soil moisture at wilting. (C) RWC plotted against soil moisture and fitted with linear plateau regression. Colors indicate individual plants. The x-value of the junction point of the regression is the soil moisture at leaf desiccation. (D) NDVI plotted against soil moisture and fitted with linear plateau regression. Colors indicate individual plants. The x-value of the junction point of the regression is the soil moisture at leaf desiccation. (E) Root/shoot ratio. A cross indicates the mean, which is used as the fifth indicator for each accession.


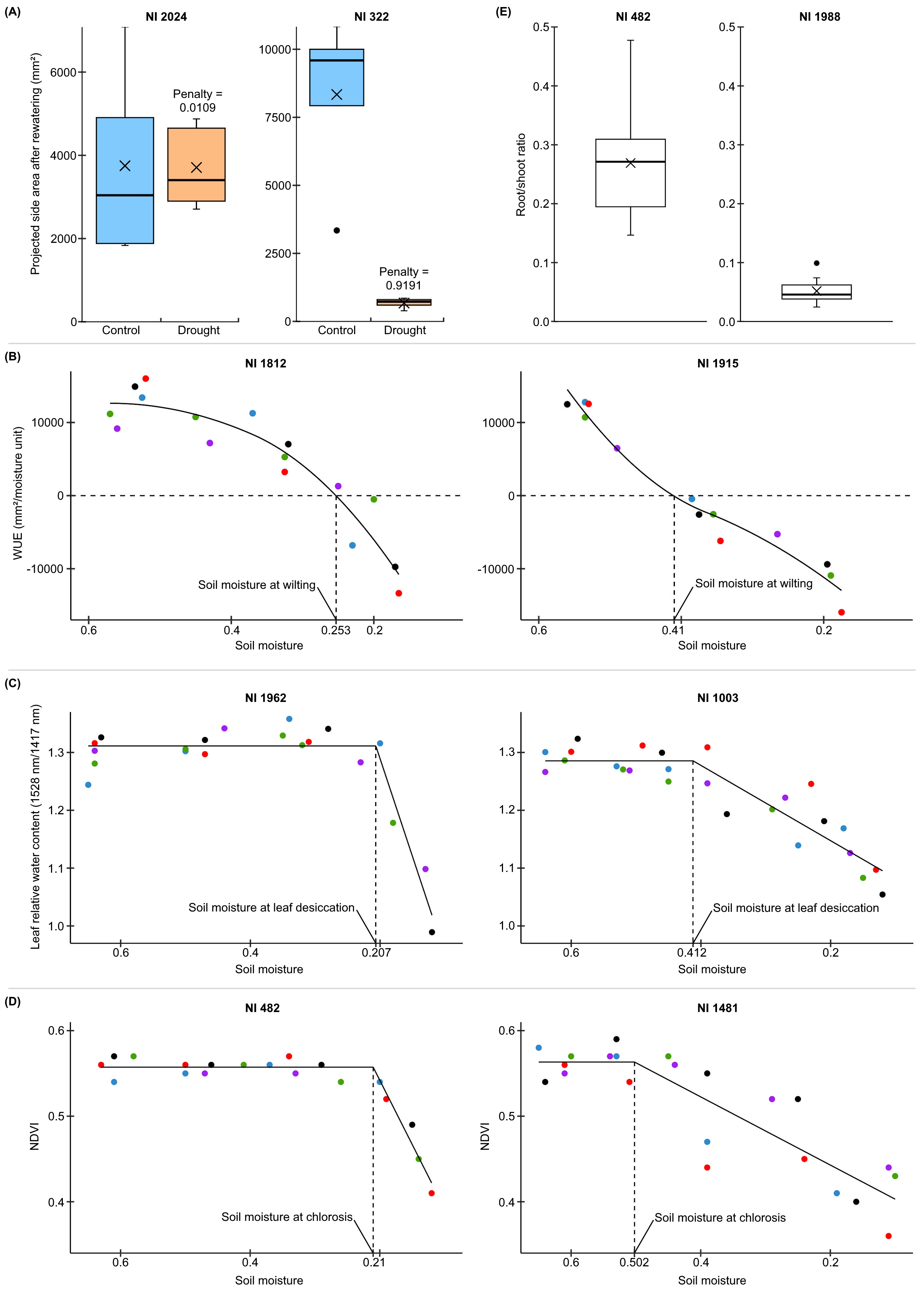


*Supplementary Figure 5.* Histogram showing the number of days after sowing (DAS) when the soil of plants under drought treatment, which started from 18 DAS, reached the threshold soil moisture value of 20%. For the extreme cases, individual accessions are listed, with the number in brackets representing the number of plants (out of five replicates) that reached the threshold value on that day. Precise data for all accessions can be found in Supplementary Table 1.


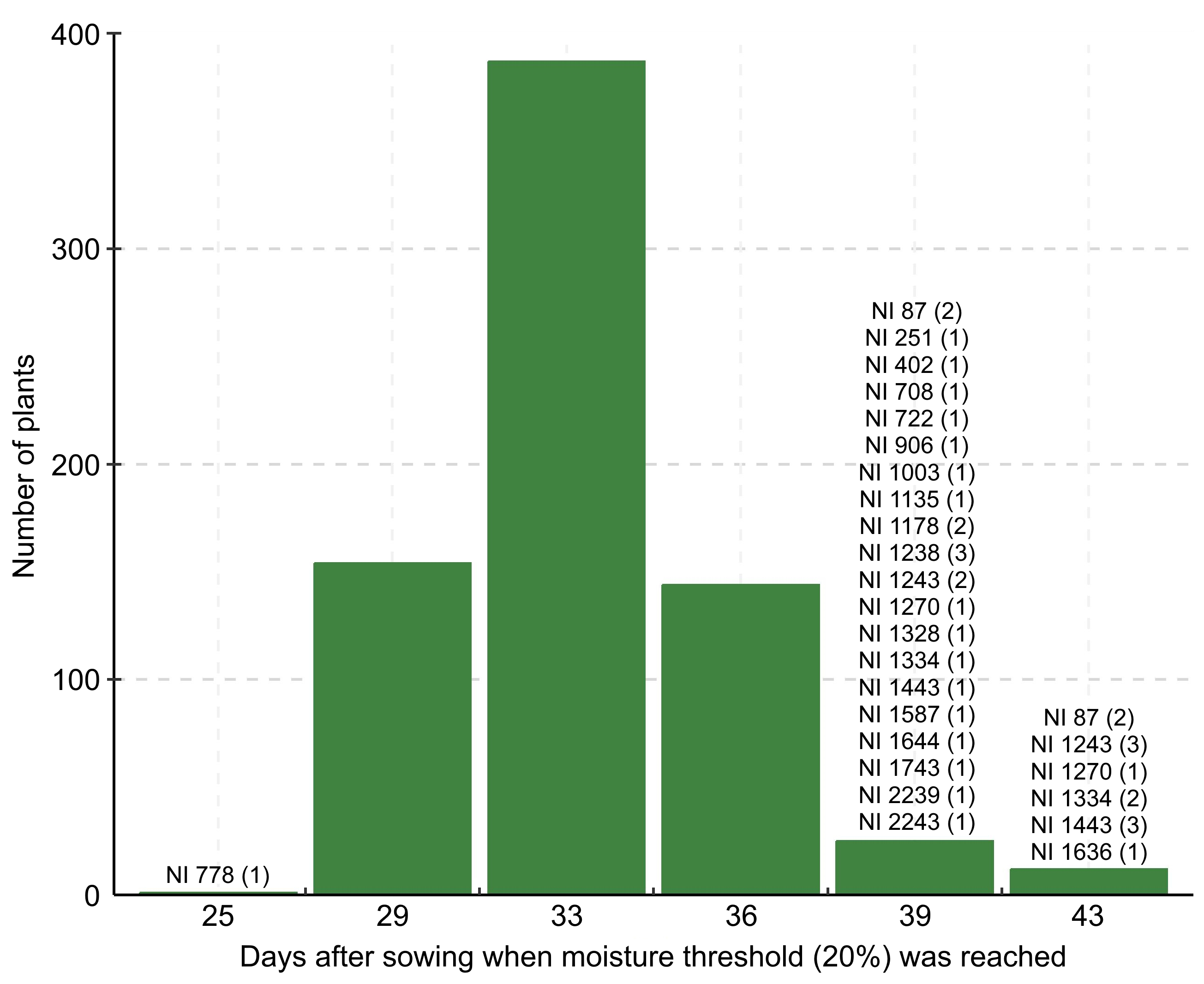


*Supplementary Figure 6.* Scatterplots of the five drought resistance indicators in relation to each other.


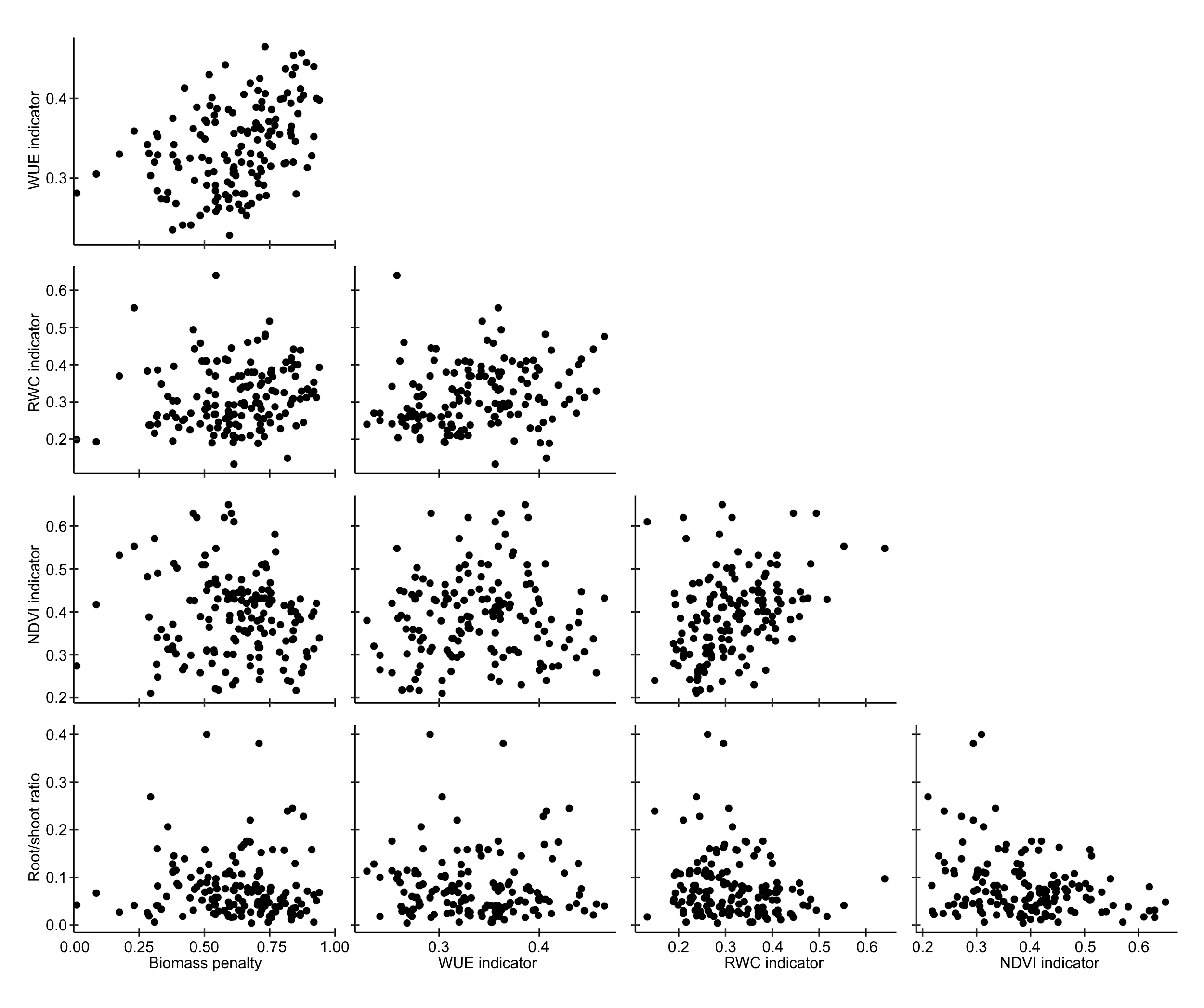


*Supplementary Figure 7.* Boxplots of median drought response indicator values for different subgenera of *Vigna* and *Phaseolus* beans (including former subgenera that were elevated to genera). Subgenera with 3 or fewer accessions were omitted. Black points indicate individual accessions. (A) Biomass penalty, (B) WUE indicator, (C) RWC indicator, (D) NDVI indicator, (E) Root/shoot ratio. N: number of accessions, SD: standard deviation, CV: coefficient of variation, *V.*: *Vigna*, *P.*: *Phaseolus*.


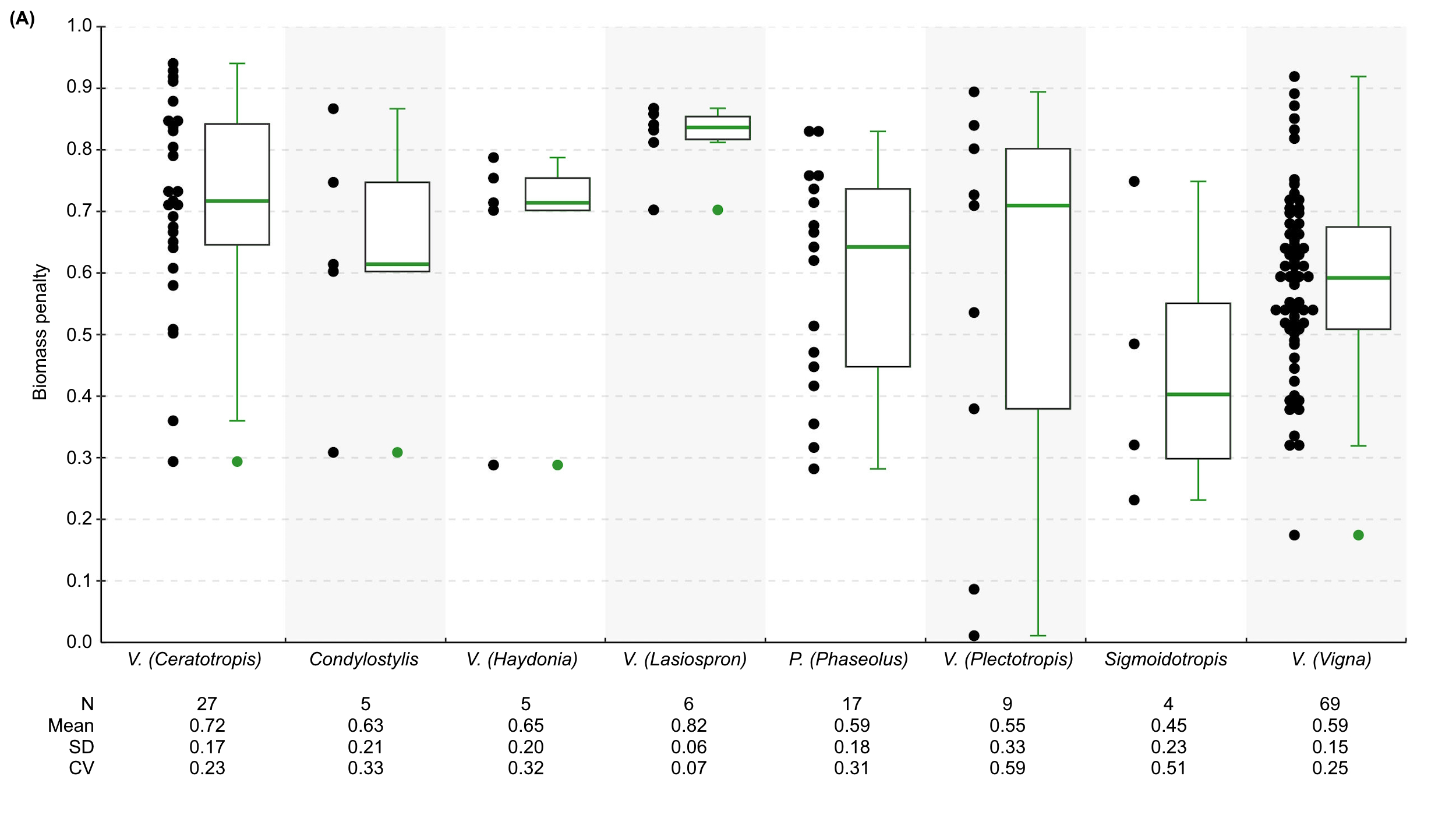


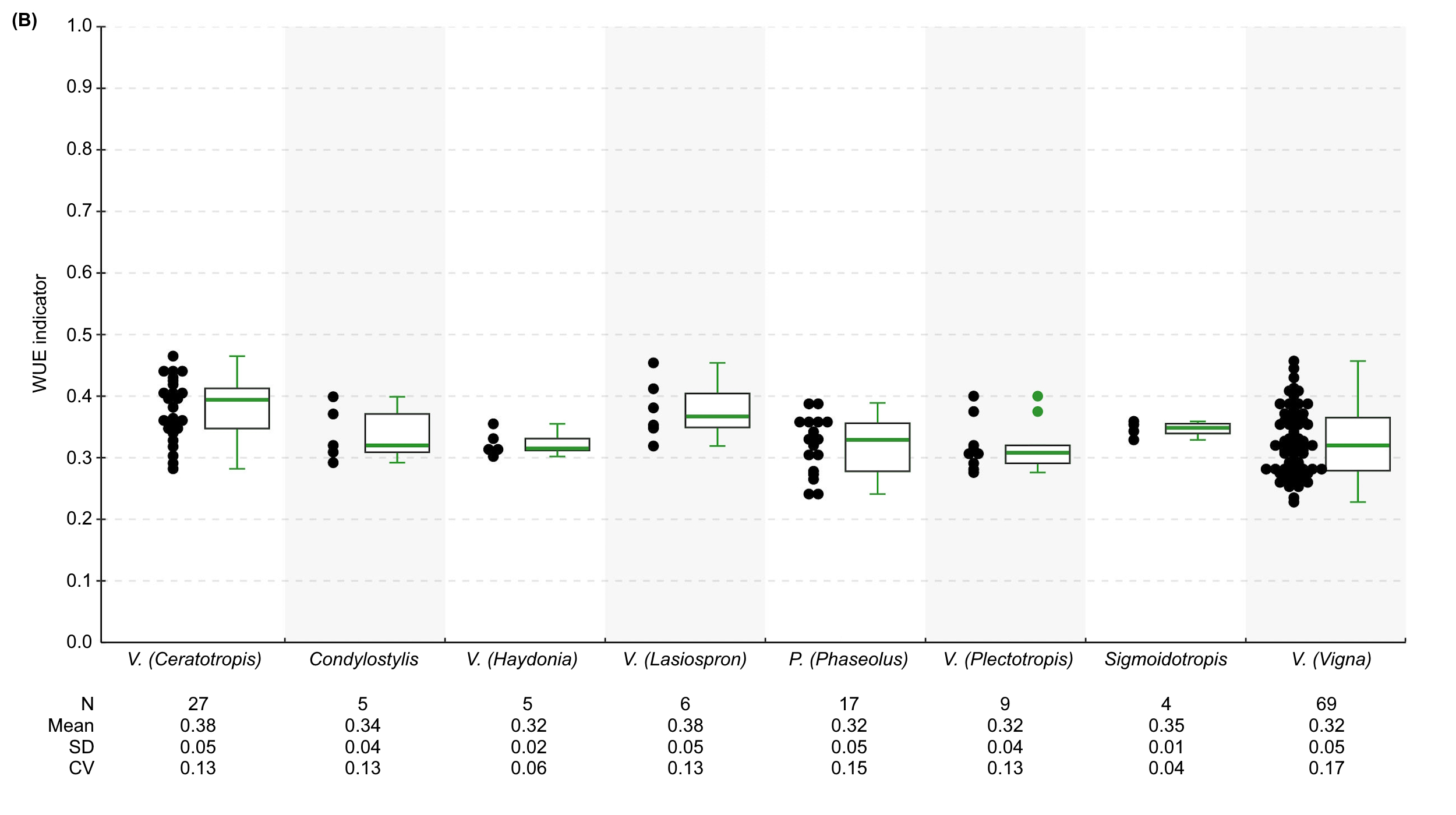


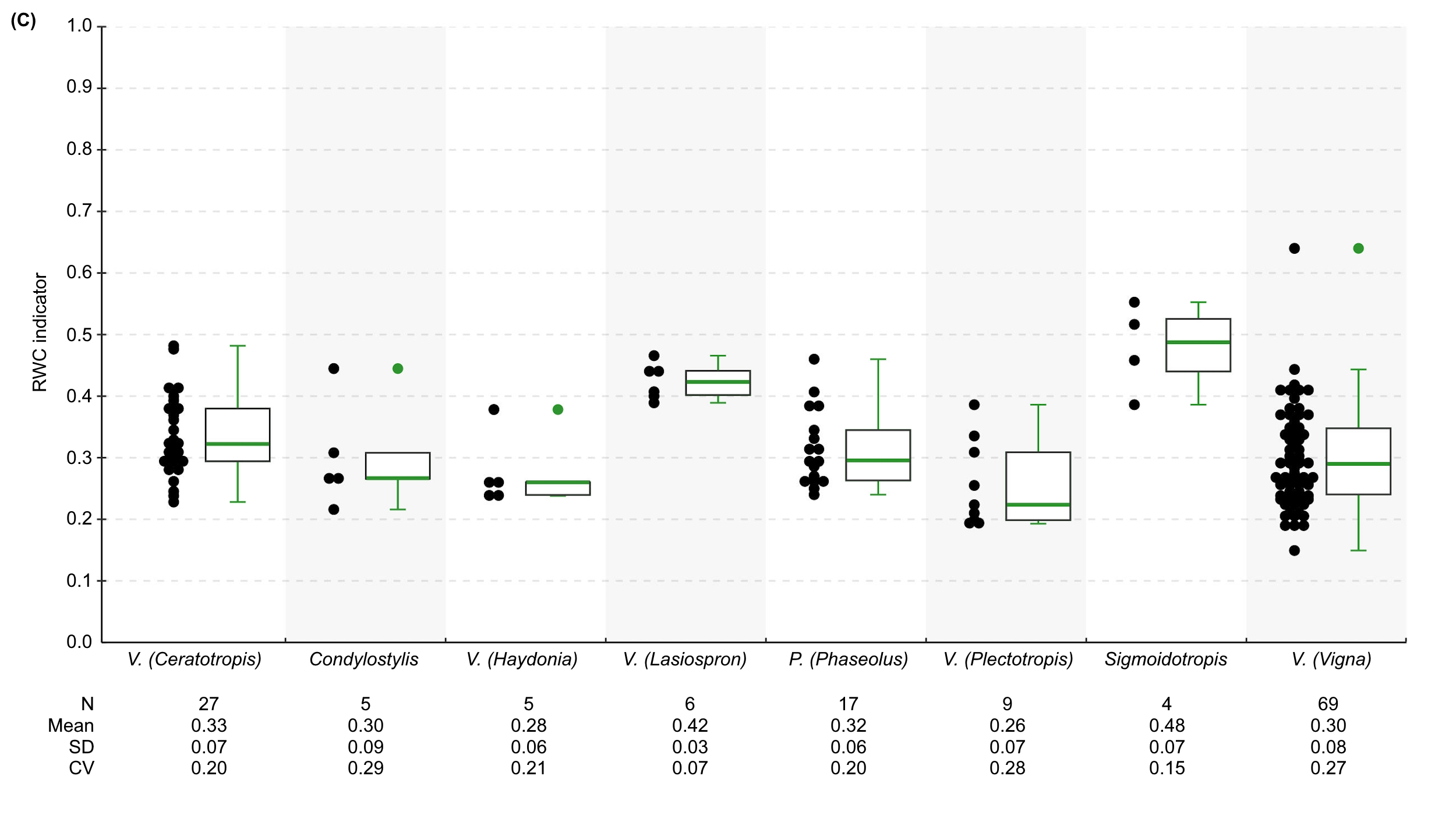


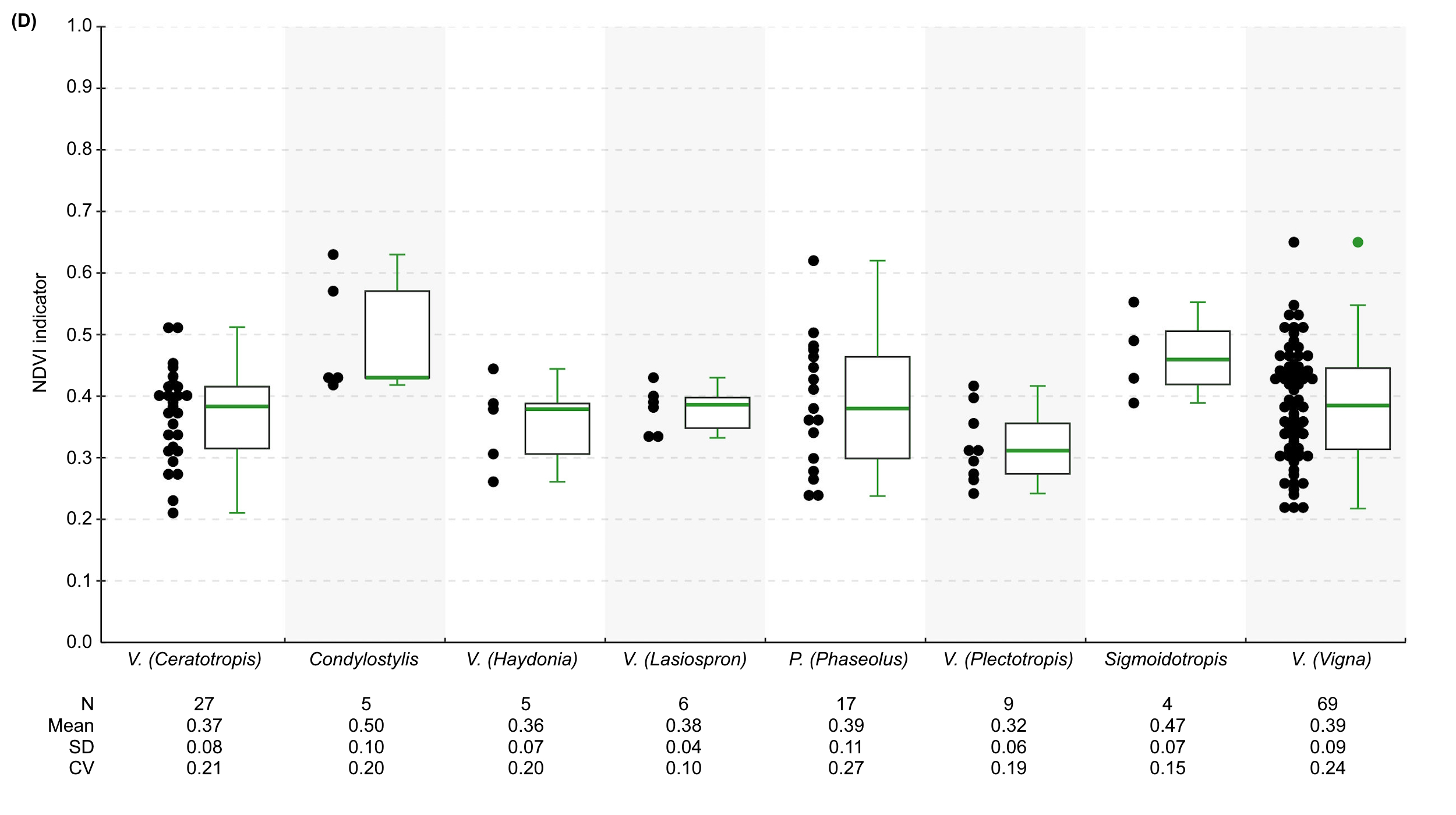


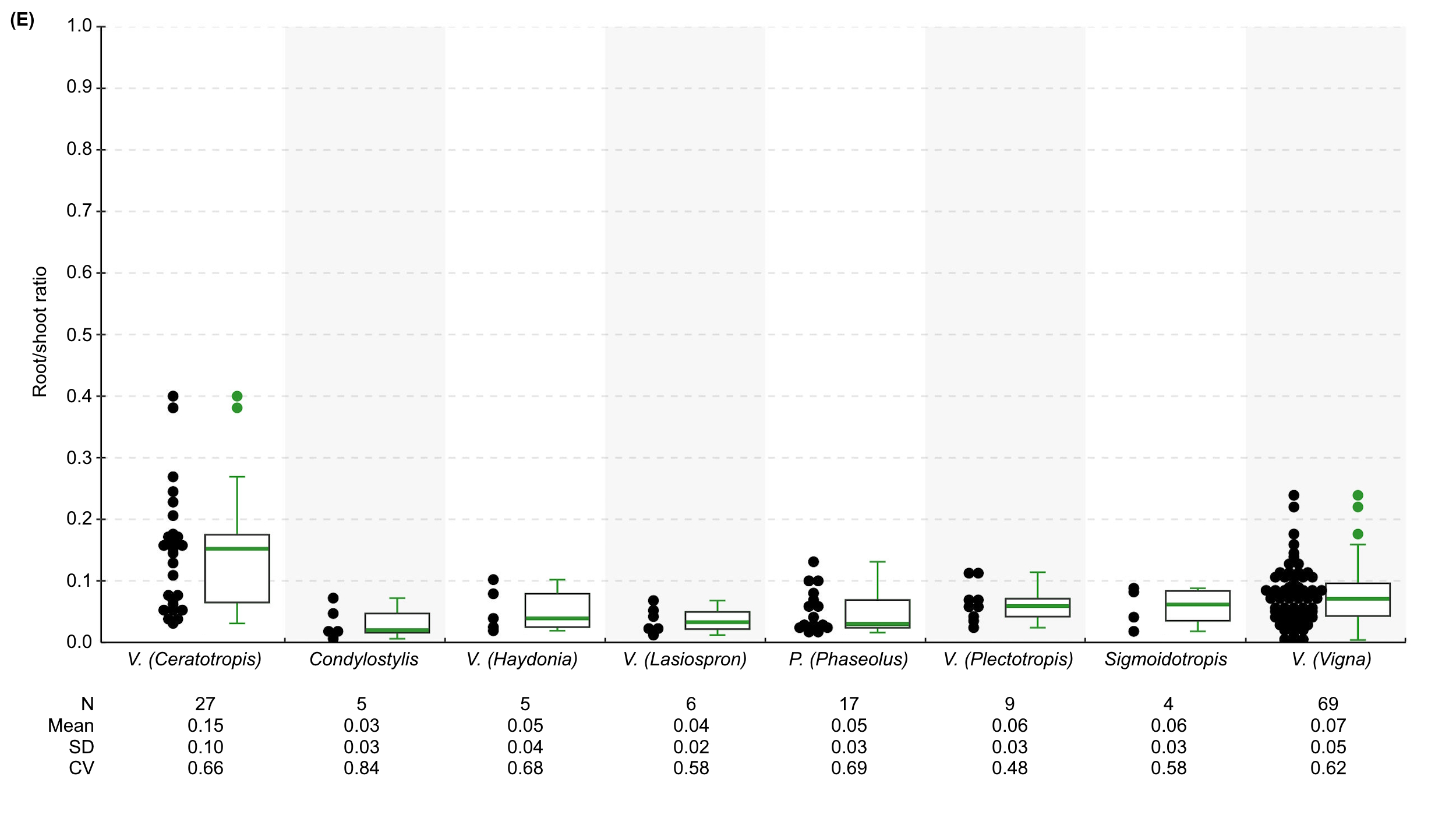


*Supplementary Figure 8.* Principal component analysis (PCA) performed with only the cultivated species (cultivated accessions + wild accessions from the same species). The wild or cultivated origin is indicated with circles or squares, respectively, while the different species are indicated with different colors.


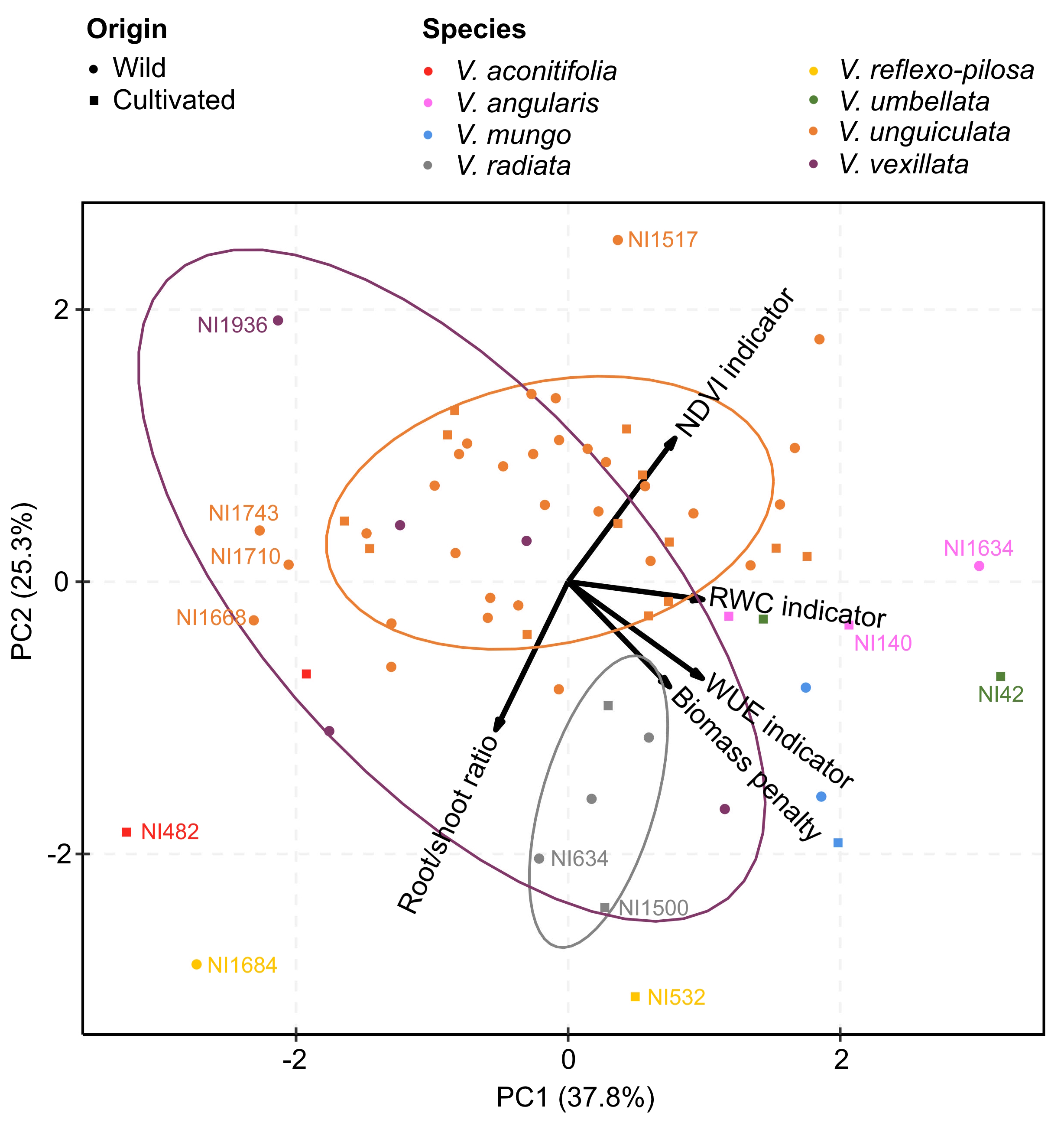


*Supplementary Figure 9.* Principal component analysis (PCA) performed with all 151 accessions to visualise the relationships between five different drought response indicators. The distribution of the wild and cultivated accessions on the first two PC axes is indicated with red and blue dots, respectively.


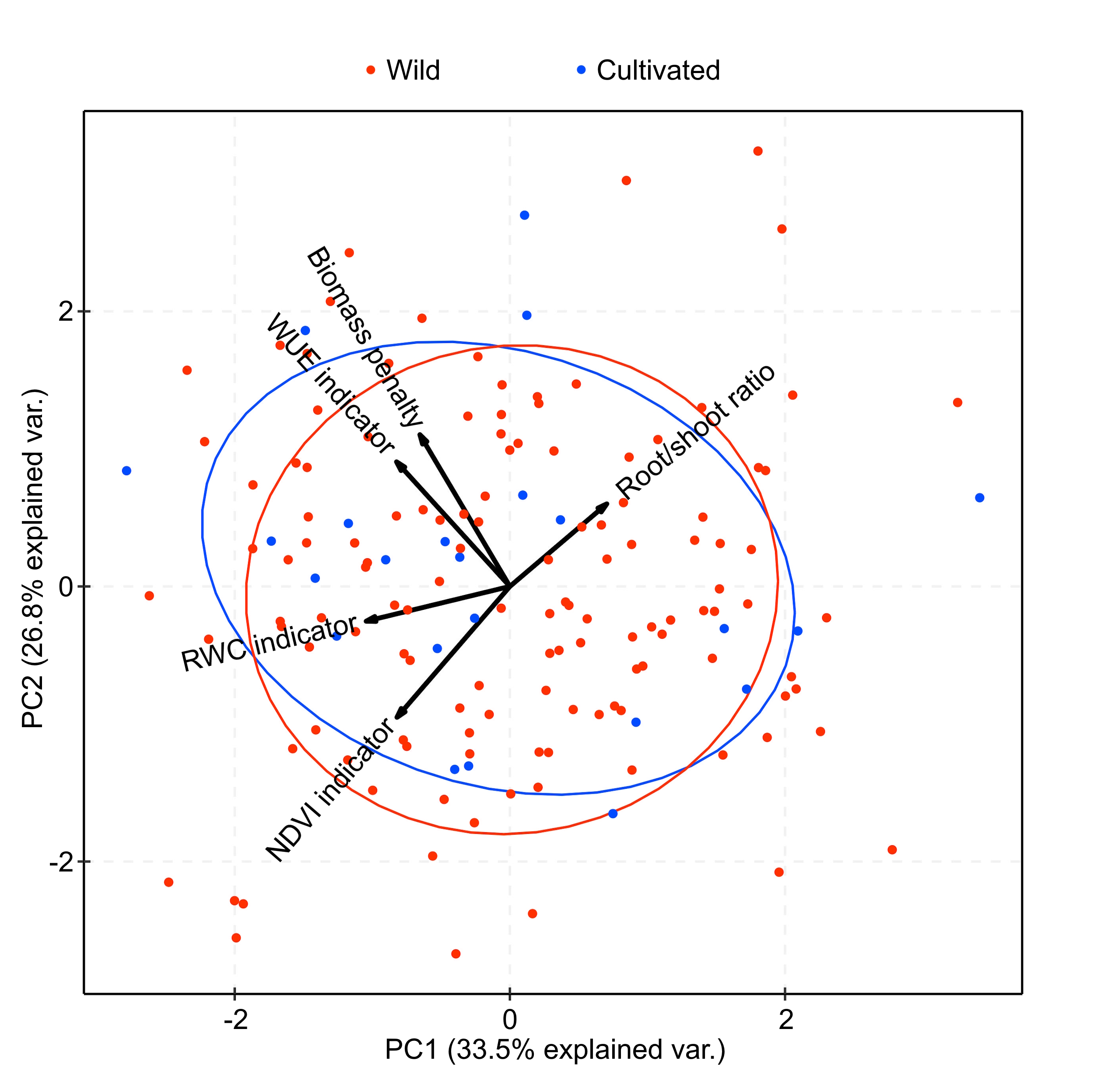

Supplement: Supplementary file 1 [file DataSheet1.docx]
